# Supplementary material for: Socio-economic-demographic determinants of depression in Indonesia: A hospital-based study
Source: PLoS One. 2020 Dec 15;15(12):e0244108. doi: 10.1371/journal.pone.0244108 (PMC7737985; doi:10.1371/journal.pone.0244108)
Supplement: S1 Appendix — (DOCX) [file pone.0244108.s001.docx]

# **S1 Appendix. Original and imputation results for socio-economic-demographic variables with missing value in Table 3.**

1. **Cross-tabulation of Case-Control and Age**

| **Case-Control * Age Cross-tabulation** | | | | | | | | |
| --- | --- | --- | --- | --- | --- | --- | --- | --- |
| Imputation Number | | | | Age | | | | Total |
|  |  |  |  | < 32 years | 32-42 years | 43-52 years | > 52 years |  |
| Original data | Case-Control | Control | Count | 43 | 51 | 33 | 33 | 160 |
|  |  |  | % within Age | 56.6% | 60.7% | 44.6% | 43.4% | 51.6% |
|  |  | Case | Count | 33 | 33 | 41 | 43 | 150 |
|  |  |  | % within Age | 43.4% | 39.3% | 55.4% | 56.6% | 48.4% |
|  | Total | | Count | 76 | 84 | 74 | 76 | 310 |
|  |  |  | % within Age | 100.0% | 100.0% | 100.0% | 100.0% | 100.0% |
| 1 | Case-Control | Control | Count | 43 | 51 | 33 | 33 | 160 |
|  |  |  | % within Age | 55.1% | 56.7% | 44.0% | 42.9% | 50.0% |
|  |  | Case | Count | 35 | 39 | 42 | 44 | 160 |
|  |  |  | % within Age | 44.9% | 43.3% | 56.0% | 57.1% | 50.0% |
|  | Total | | Count | 78 | 90 | 75 | 77 | 320 |
|  |  |  | % within Age | 100.0% | 100.0% | 100.0% | 100.0% | 100.0% |
| 2 | Case-Control | Control | Count | 43 | 51 | 33 | 33 | 160 |
|  |  |  | % within Age | 56.6% | 58.0% | 42.3% | 42.3% | 50.0% |
|  |  | Case | Count | 33 | 37 | 45 | 45 | 160 |
|  |  |  | % within Age | 43.4% | 42.0% | 57.7% | 57.7% | 50.0% |
|  | Total | | Count | 76 | 88 | 78 | 78 | 320 |
|  |  |  | % within Age | 100.0% | 100.0% | 100.0% | 100.0% | 100.0% |
| 3 | Case-Control | Control | Count | 43 | 51 | 33 | 33 | 160 |
|  |  |  | % within Age | 53.8% | 60.0% | 43.4% | 41.8% | 50.0% |
|  |  | Case | Count | 37 | 34 | 43 | 46 | 160 |
|  |  |  | % within Age | 46.3% | 40.0% | 56.6% | 58.2% | 50.0% |
|  | Total | | Count | 80 | 85 | 76 | 79 | 320 |
|  |  |  | % within Age | 100.0% | 100.0% | 100.0% | 100.0% | 100.0% |
| 4 | Case-Control | Control | Count | 43 | 51 | 33 | 33 | 160 |
|  |  |  | % within Age | 55.8% | 58.0% | 42.3% | 42.9% | 50.0% |
|  |  | Case | Count | 34 | 37 | 45 | 44 | 160 |
|  |  |  | % within Age | 44.2% | 42.0% | 57.7% | 57.1% | 50.0% |
|  | Total | | Count | 77 | 88 | 78 | 77 | 320 |
|  |  |  | % within Age | 100.0% | 100.0% | 100.0% | 100.0% | 100.0% |
| 5 | Case-Control | Control | Count | 43 | 51 | 33 | 33 | 160 |
|  |  |  | % within Age | 55.8% | 58.0% | 42.3% | 42.9% | 50.0% |
|  |  | Case | Count | 34 | 37 | 45 | 44 | 160 |
|  |  |  | % within Age | 44.2% | 42.0% | 57.7% | 57.1% | 50.0% |
|  | Total | | Count | 77 | 88 | 78 | 77 | 320 |
|  |  |  | % within Age | 100.0% | 100.0% | 100.0% | 100.0% | 100.0% |
| Pooled | Case-Control | Control | Count | 43 | 51 | 33 | 33 | 160 |
|  |  | Case | Count | 34.6 | 36.8 | 44 | 44.6 | 160 |
|  | Total | | Count | 77.6 | 87.8 | 77 | 77.6 | 320 |

1. **Cross-tabulation of Case-Control and Income**

| **Case-Control * Income Crosstabulation** | | | | | | | |
| --- | --- | --- | --- | --- | --- | --- | --- |
| Imputation Number | | | | Income | | | Total |
|  |  |  |  | High Income | Middle Income | Low Income |  |
| Original data | Case-Control | Control | Count | 33 | 59 | 40 | 132 |
|  |  |  | % within Income | 49.3% | 73.8% | 54.1% | 59.7% |
|  |  | Case | Count | 34 | 21 | 34 | 89 |
|  |  |  | % within Income | 50.7% | 26.3% | 45.9% | 40.3% |
|  | Total | | Count | 67 | 80 | 74 | 221 |
|  |  |  | % within Income | 100.0% | 100.0% | 100.0% | 100.0% |
| 1 | Case-Control | Control | Count | 45 | 72 | 43 | 160 |
|  |  |  | % within Income | 42.5% | 67.3% | 40.2% | 50.0% |
|  |  | Case | Count | 61 | 35 | 64 | 160 |
|  |  |  | % within Income | 57.5% | 32.7% | 59.8% | 50.0% |
|  | Total | | Count | 106 | 107 | 107 | 320 |
|  |  |  | % within Income | 100.0% | 100.0% | 100.0% | 100.0% |
| 2 | Case-Control | Control | Count | 41 | 66 | 53 | 160 |
|  |  |  | % within Income | 38.3% | 62.3% | 49.5% | 50.0% |
|  |  | Case | Count | 66 | 40 | 54 | 160 |
|  |  |  | % within Income | 61.7% | 37.7% | 50.5% | 50.0% |
|  | Total | | Count | 107 | 106 | 107 | 320 |
|  |  |  | % within Income | 100.0% | 100.0% | 100.0% | 100.0% |
| 3 | Case-Control | Control | Count | 44 | 68 | 48 | 160 |
|  |  |  | % within Income | 41.1% | 61.8% | 46.6% | 50.0% |
|  |  | Case | Count | 63 | 42 | 55 | 160 |
|  |  |  | % within Income | 58.9% | 38.2% | 53.4% | 50.0% |
|  | Total | | Count | 107 | 110 | 103 | 320 |
|  |  |  | % within Income | 100.0% | 100.0% | 100.0% | 100.0% |
| 4 | Case-Control | Control | Count | 44 | 71 | 45 | 160 |
|  |  |  | % within Income | 41.1% | 67.0% | 42.1% | 50.0% |
|  |  | Case | Count | 63 | 35 | 62 | 160 |
|  |  |  | % within Income | 58.9% | 33.0% | 57.9% | 50.0% |
|  | Total | | Count | 107 | 106 | 107 | 320 |
|  |  |  | % within Income | 100.0% | 100.0% | 100.0% | 100.0% |
| 5 | Case-Control | Control | Count | 45 | 63 | 52 | 160 |
|  |  |  | % within Income | 42.1% | 59.4% | 48.6% | 50.0% |
|  |  | Case | Count | 62 | 43 | 55 | 160 |
|  |  |  | % within Income | 57.9% | 40.6% | 51.4% | 50.0% |
|  | Total | | Count | 107 | 106 | 107 | 320 |
|  |  |  | % within Income | 100.0% | 100.0% | 100.0% | 100.0% |
| Pooled | Case-Control | Control | Count | 43.8 | 68 | 48.2 | 160 |
|  |  | Case | Count | 63 | 39 | 58 | 160 |
|  | Total | | Count | 106.8 | 107 | 106.2 | 320 |

1. **Cross-tabulation of Case-Control and Education**

| **Case-Control * Education Cross-tabulation** | | | | | | | |
| --- | --- | --- | --- | --- | --- | --- | --- |
| Imputation Number | | | | Education | | | Total |
|  |  |  |  | High Education | Middle Education | Low Education |  |
| Original data | Case-Control | Control | Count | 69 | 61 | 30 | 160 |
|  |  |  | % within Education | 51.9% | 44.9% | 60.0% | 50.2% |
|  |  | Case | Count | 64 | 75 | 20 | 159 |
|  |  |  | % within Education | 48.1% | 55.1% | 40.0% | 49.8% |
|  | Total | | Count | 133 | 136 | 50 | 319 |
|  |  |  | % within Education | 100.0% | 100.0% | 100.0% | 100.0% |
| 1 | Case-Control | Control | Count | 69 | 61 | 30 | 160 |
|  |  |  | % within Education | 51.5% | 44.9% | 60.0% | 50.0% |
|  |  | Case | Count | 65 | 75 | 20 | 160 |
|  |  |  | % within Education | 48.5% | 55.1% | 40.0% | 50.0% |
|  | Total | | Count | 134 | 136 | 50 | 320 |
|  |  |  | % within Education | 100.0% | 100.0% | 100.0% | 100.0% |
| 2 | Case-Control | Control | Count | 69 | 61 | 30 | 160 |
|  |  |  | % within Education | 51.5% | 44.9% | 60.0% | 50.0% |
|  |  | Case | Count | 65 | 75 | 20 | 160 |
|  |  |  | % within Education | 48.5% | 55.1% | 40.0% | 50.0% |
|  | Total | | Count | 134 | 136 | 50 | 320 |
|  |  |  | % within Education | 100.0% | 100.0% | 100.0% | 100.0% |
| 3 | Case-Control | Control | Count | 69 | 61 | 30 | 160 |
|  |  |  | % within Education | 51.5% | 44.9% | 60.0% | 50.0% |
|  |  | Case | Count | 65 | 75 | 20 | 160 |
|  |  |  | % within Education | 48.5% | 55.1% | 40.0% | 50.0% |
|  | Total | | Count | 134 | 136 | 50 | 320 |
|  |  |  | % within Education | 100.0% | 100.0% | 100.0% | 100.0% |
| 4 | Case-Control | Control | Count | 69 | 61 | 30 | 160 |
|  |  |  | % within Education | 51.9% | 44.5% | 60.0% | 50.0% |
|  |  | Case | Count | 64 | 76 | 20 | 160 |
|  |  |  | % within Education | 48.1% | 55.5% | 40.0% | 50.0% |
|  | Total | | Count | 133 | 137 | 50 | 320 |
|  |  |  | % within Education | 100.0% | 100.0% | 100.0% | 100.0% |
| 5 | Case-Control | Control | Count | 69 | 61 | 30 | 160 |
|  |  |  | % within Education | 51.5% | 44.9% | 60.0% | 50.0% |
|  |  | Case | Count | 65 | 75 | 20 | 160 |
|  |  |  | % within Education | 48.5% | 55.1% | 40.0% | 50.0% |
|  | Total | | Count | 134 | 136 | 50 | 320 |
|  |  |  | % within Education | 100.0% | 100.0% | 100.0% | 100.0% |
| Pooled | Case-Control | Control | Count | 69 | 61 | 30 | 160 |
|  |  | Case | Count | 64.8 | 75.2 | 20 | 160 |
|  | Total | | Count | 133.8 | 136.2 | 50 | 320 |

1. **Cross-tabulation of Case-Control and Occupation**

| **Case-Control * Occupation Cross-tabulation** | | | | | | | | | |
| --- | --- | --- | --- | --- | --- | --- | --- | --- | --- |
| Imputation Number | | | | Occupation | | | | | Total |
|  |  |  |  | Unemployed | Housewife | Retired | Civil Servant | Private |  |
| Original data | Case-Control | Control | Count | 7 | 48 | 3 | 19 | 82 | 159 |
|  |  |  | % within Occupation | 38.9% | 53.3% | 23.1% | 55.9% | 57.7% | 53.5% |
|  |  | Case | Count | 11 | 42 | 10 | 15 | 60 | 138 |
|  |  |  | % within Occupation | 61.1% | 46.7% | 76.9% | 44.1% | 42.3% | 46.5% |
|  | Total | | Count | 18 | 90 | 13 | 34 | 142 | 297 |
|  |  |  | % within Occupation | 100.0% | 100.0% | 100.0% | 100.0% | 100.0% | 100.0% |
| 1 | Case-Control | Control | Count | 8 | 48 | 3 | 19 | 82 | 160 |
|  |  |  | % within Occupation | 40.0% | 50.5% | 20.0% | 51.4% | 53.6% | 50.0% |
|  |  | Case | Count | 12 | 47 | 12 | 18 | 71 | 160 |
|  |  |  | % within Occupation | 60.0% | 49.5% | 80.0% | 48.6% | 46.4% | 50.0% |
|  | Total | | Count | 20 | 95 | 15 | 37 | 153 | 320 |
|  |  |  | % within Occupation | 100.0% | 100.0% | 100.0% | 100.0% | 100.0% | 100.0% |
| 2 | Case-Control | Control | Count | 7 | 49 | 3 | 19 | 82 | 160 |
|  |  |  | % within Occupation | 35.0% | 50.5% | 23.1% | 51.4% | 53.6% | 50.0% |
|  |  | Case | Count | 13 | 48 | 10 | 18 | 71 | 160 |
|  |  |  | % within Occupation | 65.0% | 49.5% | 76.9% | 48.6% | 46.4% | 50.0% |
|  | Total | | Count | 20 | 97 | 13 | 37 | 153 | 320 |
|  |  |  | % within Occupation | 100.0% | 100.0% | 100.0% | 100.0% | 100.0% | 100.0% |
| 3 | Case-Control | Control | Count | 7 | 49 | 3 | 19 | 82 | 160 |
|  |  |  | % within Occupation | 36.8% | 51.0% | 20.0% | 52.8% | 53.2% | 50.0% |
|  |  | Case | Count | 12 | 47 | 12 | 17 | 72 | 160 |
|  |  |  | % within Occupation | 63.2% | 49.0% | 80.0% | 47.2% | 46.8% | 50.0% |
|  | Total | | Count | 19 | 96 | 15 | 36 | 154 | 320 |
|  |  |  | % within Occupation | 100.0% | 100.0% | 100.0% | 100.0% | 100.0% | 100.0% |
| 4 | Case-Control | Control | Count | 7 | 49 | 3 | 19 | 82 | 160 |
|  |  |  | % within Occupation | 33.3% | 51.6% | 23.1% | 54.3% | 52.6% | 50.0% |
|  |  | Case | Count | 14 | 46 | 10 | 16 | 74 | 160 |
|  |  |  | % within Occupation | 66.7% | 48.4% | 76.9% | 45.7% | 47.4% | 50.0% |
|  | Total | | Count | 21 | 95 | 13 | 35 | 156 | 320 |
|  |  |  | % within Occupation | 100.0% | 100.0% | 100.0% | 100.0% | 100.0% | 100.0% |
| 5 | Case-Control | Control | Count | 8 | 48 | 3 | 19 | 82 | 160 |
|  |  |  | % within Occupation | 40.0% | 51.1% | 21.4% | 51.4% | 52.9% | 50.0% |
|  |  | Case | Count | 12 | 46 | 11 | 18 | 73 | 160 |
|  |  |  | % within Occupation | 60.0% | 48.9% | 78.6% | 48.6% | 47.1% | 50.0% |
|  | Total | | Count | 20 | 94 | 14 | 37 | 155 | 320 |
|  |  |  | % within Occupation | 100.0% | 100.0% | 100.0% | 100.0% | 100.0% | 100.0% |
| Pooled | Case-Control | Control | Count | 7.4 | 48.6 | 3 | 19 | 82 | 160 |
|  |  | Case | Count | 12.6 | 46.8 | 11 | 17.4 | 72.2 | 160 |
|  | Total | | Count | 20 | 95.4 | 14 | 36.4 | 154.2 | 320 |

1. **Cross-tabulation of Case-Control and Hours Worked**

| **Case-Control * Hours Worked Cross-tabulation** | | | | | | |
| --- | --- | --- | --- | --- | --- | --- |
| Imputation Number | | | | Hours Worked (dichotom) | | Total |
|  |  |  |  | < 6 hours | >= 6 hours |  |
| Original data | Case-Control | Control | Count | 66 | 94 | 160 |
|  |  |  | % within Hours Worked | 53.7% | 53.4% | 53.5% |
|  |  | Case | Count | 57 | 82 | 139 |
|  |  |  | % within Hours Worked | 46.3% | 46.6% | 46.5% |
|  | Total | | Count | 123 | 176 | 299 |
|  |  |  | % within Hours Worked | 100.0% | 100.0% | 100.0% |
| 1 | Case-Control | Control | Count | 66 | 94 | 160 |
|  |  |  | % within Hours Worked | 48.9% | 50.8% | 50.0% |
|  |  | Case | Count | 69 | 91 | 160 |
|  |  |  | % within Hours Worked | 51.1% | 49.2% | 50.0% |
|  | Total | | Count | 135 | 185 | 320 |
|  |  |  | % within Hours Worked | 100.0% | 100.0% | 100.0% |
| 2 | Case-Control | Control | Count | 66 | 94 | 160 |
|  |  |  | % within Hours Worked | 49.3% | 50.5% | 50.0% |
|  |  | Case | Count | 68 | 92 | 160 |
|  |  |  | % within Hours Worked | 50.7% | 49.5% | 50.0% |
|  | Total | | Count | 134 | 186 | 320 |
|  |  |  | % within Hours Worked | 100.0% | 100.0% | 100.0% |
| 3 | Case-Control | Control | Count | 66 | 94 | 160 |
|  |  |  | % within Hours Worked | 48.2% | 51.4% | 50.0% |
|  |  | Case | Count | 71 | 89 | 160 |
|  |  |  | % within Hours Worked | 51.8% | 48.6% | 50.0% |
|  | Total | | Count | 137 | 183 | 320 |
|  |  |  | % within Hours Worked | 100.0% | 100.0% | 100.0% |
| 4 | Case-Control | Control | Count | 66 | 94 | 160 |
|  |  |  | % within Hours Worked | 48.5% | 51.1% | 50.0% |
|  |  | Case | Count | 70 | 90 | 160 |
|  |  |  | % within Hours Worked | 51.5% | 48.9% | 50.0% |
|  | Total | | Count | 136 | 184 | 320 |
|  |  |  | % within Hours Worked | 100.0% | 100.0% | 100.0% |
| 5 | Case-Control | Control | Count | 66 | 94 | 160 |
|  |  |  | % within Hours Worked | 47.8% | 51.6% | 50.0% |
|  |  | Case | Count | 72 | 88 | 160 |
|  |  |  | % within Hours Worked | 52.2% | 48.4% | 50.0% |
|  | Total | | Count | 138 | 182 | 320 |
|  |  |  | % within Hours Worked | 100.0% | 100.0% | 100.0% |
| Pooled | Case-Control | Control | Count | 66 | 94 | 160 |
|  |  | Case | Count | 70 | 90 | 160 |
|  | Total | | Count | 136 | 184 | 320 |

1. **Cross-tabulation of Case-Control and Cohabitating family members**

| **Case-Control * Cohabitating family members Cross-tabulation** | | | | | | | |
| --- | --- | --- | --- | --- | --- | --- | --- |
| Imputation Number | | | | Cohabitating family members | | | Total |
|  |  |  |  | with 0 | with 1-3 | with >=4 |  |
| Original data | Case-Control | Control | Count | 12 | 86 | 62 | 160 |
|  |  |  | % within Cohabitating family members | 63.2% | 54.4% | 44.0% | 50.3% |
|  |  | Case | Count | 7 | 72 | 79 | 158 |
|  |  |  | % within Cohabitating family members | 36.8% | 45.6% | 56.0% | 49.7% |
|  | Total | | Count | 19 | 158 | 141 | 318 |
|  |  |  | % within Cohabitating family members | 100.0% | 100.0% | 100.0% | 100.0% |
| 1 | Case-Control | Control | Count | 12 | 86 | 62 | 160 |
|  |  |  | % within Cohabitating family members | 63.2% | 54.4% | 43.4% | 50.0% |
|  |  | Case | Count | 7 | 72 | 81 | 160 |
|  |  |  | % within Cohabitating family members | 36.8% | 45.6% | 56.6% | 50.0% |
|  | Total | | Count | 19 | 158 | 143 | 320 |
|  |  |  | % within Cohabitating family members | 100.0% | 100.0% | 100.0% | 100.0% |
| 2 | Case-Control | Control | Count | 12 | 86 | 62 | 160 |
|  |  |  | % within Cohabitating family members | 63.2% | 54.4% | 43.4% | 50.0% |
|  |  | Case | Count | 7 | 72 | 81 | 160 |
|  |  |  | % within Cohabitating family members | 36.8% | 45.6% | 56.6% | 50.0% |
|  | Total | | Count | 19 | 158 | 143 | 320 |
|  |  |  | % within Cohabitating family members | 100.0% | 100.0% | 100.0% | 100.0% |
| 3 | Case-Control | Control | Count | 12 | 86 | 62 | 160 |
|  |  |  | % within Cohabitating family members | 63.2% | 54.1% | 43.7% | 50.0% |
|  |  | Case | Count | 7 | 73 | 80 | 160 |
|  |  |  | % within Cohabitating family members | 36.8% | 45.9% | 56.3% | 50.0% |
|  | Total | | Count | 19 | 159 | 142 | 320 |
|  |  |  | % within Number of Family Living with | 100.0% | 100.0% | 100.0% | 100.0% |
| 4 | Case-Control | Control | Count | 12 | 86 | 62 | 160 |
|  |  |  | % within Cohabitating family members | 63.2% | 54.1% | 44.0% | 50.2% |
|  |  | Case | Count | 7 | 73 | 79 | 159 |
|  |  |  | % within Cohabitating family members | 36.8% | 45.9% | 56.0% | 49.8% |
|  | Total | | Count | 19 | 159 | 141 | 319 |
|  |  |  | % within Cohabitating family members | 100.0% | 100.0% | 100.0% | 100.0% |
| 5 | Case-Control | Control | Count | 12 | 86 | 62 | 160 |
|  |  |  | % within Cohabitating family members | 63.2% | 53.8% | 44.0% | 50.0% |
|  |  | Case | Count | 7 | 74 | 79 | 160 |
|  |  |  | % within Cohabitating family members | 36.8% | 46.3% | 56.0% | 50.0% |
|  | Total | | Count | 19 | 160 | 141 | 320 |
|  |  |  | % within Cohabitating family members | 100.0% | 100.0% | 100.0% | 100.0% |
| Pooled | Case-Control | Control | Count | 12 | 86 | 62 | 160 |
|  |  | Case | Count | 7 | 72.8 | 80 | 159.8 |
|  | Total | | Count | 19 | 158.8 | 142 | 319.8 |
