# Supplementary material for: Socio-economic-demographic determinants of depression in Indonesia: A hospital-based study
Source: PLoS One. 2020 Dec 15;15(12):e0244108. doi: 10.1371/journal.pone.0244108 (PMC7737985; doi:10.1371/journal.pone.0244108)
Supplement: S2 Appendix — (DOCX) [file pone.0244108.s002.docx]

**S2 Appendix. Original and imputation results for socio-economic-demographic variables with missing value (income) in Table 4**

| **Income (dichotom) * Case-Control Crosstabulation** | | | | | | |
| --- | --- | --- | --- | --- | --- | --- |
| Imputation Number | | | | Case-Control | | Total |
|  |  |  |  | Control | Case |  |
| Original data | Income (dichotom) | Lower Middle Income | Count | 99 | 55 | 154 |
|  |  |  | % within Case-Control | 75.0% | 61.8% | 69.7% |
|  |  | High Income | Count | 33 | 34 | 67 |
|  |  |  | % within Case-Control | 25.0% | 38.2% | 30.3% |
|  | Total | | Count | 132 | 89 | 221 |
|  |  |  | % within Case-Control | 100.0% | 100.0% | 100.0% |
| 1 | Income (dichotom) | Lower Middle Income | Count | 115 | 99 | 214 |
|  |  |  | % within Case-Control | 71.9% | 61.9% | 66.9% |
|  |  | High Income | Count | 45 | 61 | 106 |
|  |  |  | % within Case-Control | 28.1% | 38.1% | 33.1% |
|  | Total | | Count | 160 | 160 | 320 |
|  |  |  | % within Case-Control | 100.0% | 100.0% | 100.0% |
| 2 | Income (dichotom) | Lower Middle Income | Count | 119 | 94 | 213 |
|  |  |  | % within Case-Control | 74.4% | 58.8% | 66.6% |
|  |  | High Income | Count | 41 | 66 | 107 |
|  |  |  | % within Case-Control | 25.6% | 41.3% | 33.4% |
|  | Total | | Count | 160 | 160 | 320 |
|  |  |  | % within Case-Control | 100.0% | 100.0% | 100.0% |
| 3 | Income (dichotom) | Lower Middle Income | Count | 116 | 97 | 213 |
|  |  |  | % within Case-Control | 72.5% | 60.6% | 66.6% |
|  |  | High Income | Count | 44 | 63 | 107 |
|  |  |  | % within Case-Control | 27.5% | 39.4% | 33.4% |
|  | Total | | Count | 160 | 160 | 320 |
|  |  |  | % within Case-Control | 100.0% | 100.0% | 100.0% |
| 4 | Income (dichotom) | Lower Middle Income | Count | 116 | 97 | 213 |
|  |  |  | % within Case-Control | 72.5% | 60.6% | 66.6% |
|  |  | High Income | Count | 44 | 63 | 107 |
|  |  |  | % within Case-Control | 27.5% | 39.4% | 33.4% |
|  | Total | | Count | 160 | 160 | 320 |
|  |  |  | % within Case-Control | 100.0% | 100.0% | 100.0% |
| 5 | Income (dichotom) | Lower Middle Income | Count | 115 | 98 | 213 |
|  |  |  | % within Case-Control | 71.9% | 61.3% | 66.6% |
|  |  | High Income | Count | 45 | 62 | 107 |
|  |  |  | % within Case-Control | 28.1% | 38.8% | 33.4% |
|  | Total | | Count | 160 | 160 | 320 |
|  |  |  | % within Case-Control | 100.0% | 100.0% | 100.0% |
| Pooled | Income (dichotom) | Lower Middle Income | Count | 116.2 | 97 | 213.2 |
|  |  | High Income | Count | 43.8 | 63 | 106.8 |
|  | Total | | Count | 160 | 160 | 320 |

**Mantel-Haenszel Common OR Estimate Test (for Pooled Analysis)**

| **Mantel-Haenszel Common Odds Ratio Estimate** | | | |
| --- | --- | --- | --- |
| Estimate | | | 1.723 |
| ln(Estimate) | | | .544 |
| Standard Error of ln(Estimate) | | | .240 |
| Asymptotic Significance (2-sided) | | | .023 |
| Asymptotic 95% Confidence Interval | Common Odds Ratio | Lower Bound | 1.076 |
|  |  | Upper Bound | 2.758 |
|  | ln(Common Odds Ratio) | Lower Bound | .074 |
|  |  | Upper Bound | 1.015 |
| The Mantel-Haenszel common odds ratio estimate is asymptotically normally distributed under the common odds ratio of 1.000 assumption. So is the natural log of the estimate. | | | |
