# Supplementary material for: Socio-economic-demographic determinants of depression in Indonesia: A hospital-based study
Source: PLoS One. 2020 Dec 15;15(12):e0244108. doi: 10.1371/journal.pone.0244108 (PMC7737985; doi:10.1371/journal.pone.0244108)
Supplement: S3 Appendix — (DOCX) [file pone.0244108.s003.docx]

# **S3 Appendix. Original and imputation results for socio-economic-demographic variables with missing values in Table 5**

1. **Cross-tabulation of Case-Control and Income break down by Age**

| **Case-Control * Income (dichotom) * Age Cross-tabulation** | | | | | | | |
| --- | --- | --- | --- | --- | --- | --- | --- |
| Imputation Number | Age | | | | Income (dichotom) | | Total |
|  |  |  |  |  | High Income | Lower Middle Income |  |
| Original data | < 32 years | Case-Control | Control | Count | 14 | 26 | 40 |
|  |  |  |  | % within Case-Control | 35.0% | 65.0% | 100.0% |
|  |  |  | Case | Count | 6 | 11 | 17 |
|  |  |  |  | % within Case-Control | 35.3% | 64.7% | 100.0% |
|  |  | Total | | Count | 20 | 37 | 57 |
|  |  |  |  | % within Case-Control | 35.1% | 64.9% | 100.0% |
|  | 32-42 years | Case-Control | Control | Count | 11 | 33 | 44 |
|  |  |  |  | % within Case-Control | 25.0% | 75.0% | 100.0% |
|  |  |  | Case | Count | 5 | 9 | 14 |
|  |  |  |  | % within Case-Control | 35.7% | 64.3% | 100.0% |
|  |  | Total | | Count | 16 | 42 | 58 |
|  |  |  |  | % within Case-Control | 27.6% | 72.4% | 100.0% |
|  | 43-52 years | Case-Control | Control | Count | 4 | 19 | 23 |
|  |  |  |  | % within Case-Control | 17.4% | 82.6% | 100.0% |
|  |  |  | Case | Count | 8 | 16 | 24 |
|  |  |  |  | % within Case-Control | 33.3% | 66.7% | 100.0% |
|  |  | Total | | Count | 12 | 35 | 47 |
|  |  |  |  | % within Case-Control | 25.5% | 74.5% | 100.0% |
|  | > 52 years | Case-Control | Control | Count | 4 | 21 | 25 |
|  |  |  |  | % within Case-Control | 16.0% | 84.0% | 100.0% |
|  |  |  | Case | Count | 13 | 16 | 29 |
|  |  |  |  | % within Case-Control | 44.8% | 55.2% | 100.0% |
|  |  | Total | | Count | 17 | 37 | 54 |
|  |  |  |  | % within Case-Control | 31.5% | 68.5% | 100.0% |
|  | Total | Case-Control | Control | Count | 33 | 99 | 132 |
|  |  |  |  | % within Case-Control | 25.0% | 75.0% | 100.0% |
|  |  |  | Case | Count | 32 | 52 | 84 |
|  |  |  |  | % within Case-Control | 38.1% | 61.9% | 100.0% |
|  |  | Total | | Count | 65 | 151 | 216 |
|  |  |  |  | % within Case-Control | 30.1% | 69.9% | 100.0% |
| 1 | < 32 years | Case-Control | Control | Count | 16 | 27 | 43 |
|  |  |  |  | % within Case-Control | 37.2% | 62.8% | 100.0% |
|  |  |  | Case | Count | 15 | 20 | 35 |
|  |  |  |  | % within Case-Control | 42.9% | 57.1% | 100.0% |
|  |  | Total | | Count | 31 | 47 | 78 |
|  |  |  |  | % within Case-Control | 39.7% | 60.3% | 100.0% |
|  | 32-42 years | Case-Control | Control | Count | 15 | 36 | 51 |
|  |  |  |  | % within Case-Control | 29.4% | 70.6% | 100.0% |
|  |  |  | Case | Count | 12 | 27 | 39 |
|  |  |  |  | % within Case-Control | 30.8% | 69.2% | 100.0% |
|  |  | Total | | Count | 27 | 63 | 90 |
|  |  |  |  | % within Case-Control | 30.0% | 70.0% | 100.0% |
|  | 43-52 years | Case-Control | Control | Count | 7 | 26 | 33 |
|  |  |  |  | % within Case-Control | 21.2% | 78.8% | 100.0% |
|  |  |  | Case | Count | 16 | 26 | 42 |
|  |  |  |  | % within Case-Control | 38.1% | 61.9% | 100.0% |
|  |  | Total | | Count | 23 | 52 | 75 |
|  |  |  |  | % within Case-Control | 30.7% | 69.3% | 100.0% |
|  | > 52 years | Case-Control | Control | Count | 7 | 26 | 33 |
|  |  |  |  | % within Case-Control | 21.2% | 78.8% | 100.0% |
|  |  |  | Case | Count | 18 | 26 | 44 |
|  |  |  |  | % within Case-Control | 40.9% | 59.1% | 100.0% |
|  |  | Total | | Count | 25 | 52 | 77 |
|  |  |  |  | % within Case-Control | 32.5% | 67.5% | 100.0% |
|  | Total | Case-Control | Control | Count | 45 | 115 | 160 |
|  |  |  |  | % within Case-Control | 28.1% | 71.9% | 100.0% |
|  |  |  | Case | Count | 61 | 99 | 160 |
|  |  |  |  | % within Case-Control | 38.1% | 61.9% | 100.0% |
|  |  | Total | | Count | 106 | 214 | 320 |
|  |  |  |  | % within Case-Control | 33.1% | 66.9% | 100.0% |
| 2 | < 32 years | Case-Control | Control | Count | 14 | 29 | 43 |
|  |  |  |  | % within Case-Control | 32.6% | 67.4% | 100.0% |
|  |  |  | Case | Count | 14 | 19 | 33 |
|  |  |  |  | % within Case-Control | 42.4% | 57.6% | 100.0% |
|  |  | Total | | Count | 28 | 48 | 76 |
|  |  |  |  | % within Case-Control | 36.8% | 63.2% | 100.0% |
|  | 32-42 years | Case-Control | Control | Count | 15 | 36 | 51 |
|  |  |  |  | % within Case-Control | 29.4% | 70.6% | 100.0% |
|  |  |  | Case | Count | 14 | 23 | 37 |
|  |  |  |  | % within Case-Control | 37.8% | 62.2% | 100.0% |
|  |  | Total | | Count | 29 | 59 | 88 |
|  |  |  |  | % within Case-Control | 33.0% | 67.0% | 100.0% |
|  | 43-52 years | Case-Control | Control | Count | 7 | 26 | 33 |
|  |  |  |  | % within Case-Control | 21.2% | 78.8% | 100.0% |
|  |  |  | Case | Count | 16 | 29 | 45 |
|  |  |  |  | % within Case-Control | 35.6% | 64.4% | 100.0% |
|  |  | Total | | Count | 23 | 55 | 78 |
|  |  |  |  | % within Case-Control | 29.5% | 70.5% | 100.0% |
|  | > 52 years | Case-Control | Control | Count | 5 | 28 | 33 |
|  |  |  |  | % within Case-Control | 15.2% | 84.8% | 100.0% |
|  |  |  | Case | Count | 22 | 23 | 45 |
|  |  |  |  | % within Case-Control | 48.9% | 51.1% | 100.0% |
|  |  | Total | | Count | 27 | 51 | 78 |
|  |  |  |  | % within Case-Control | 34.6% | 65.4% | 100.0% |
|  | Total | Case-Control | Control | Count | 41 | 119 | 160 |
|  |  |  |  | % within Case-Control | 25.6% | 74.4% | 100.0% |
|  |  |  | Case | Count | 66 | 94 | 160 |
|  |  |  |  | % within Case-Control | 41.3% | 58.8% | 100.0% |
|  |  | Total | | Count | 107 | 213 | 320 |
|  |  |  |  | % within Case-Control | 33.4% | 66.6% | 100.0% |
| 3 | < 32 years | Case-Control | Control | Count | 15 | 28 | 43 |
|  |  |  |  | % within Case-Control | 34.9% | 65.1% | 100.0% |
|  |  |  | Case | Count | 15 | 22 | 37 |
|  |  |  |  | % within Case-Control | 40.5% | 59.5% | 100.0% |
|  |  | Total | | Count | 30 | 50 | 80 |
|  |  |  |  | % within Case-Control | 37.5% | 62.5% | 100.0% |
|  | 32-42 years | Case-Control | Control | Count | 14 | 37 | 51 |
|  |  |  |  | % within Case-Control | 27.5% | 72.5% | 100.0% |
|  |  |  | Case | Count | 13 | 21 | 34 |
|  |  |  |  | % within Case-Control | 38.2% | 61.8% | 100.0% |
|  |  | Total | | Count | 27 | 58 | 85 |
|  |  |  |  | % within Case-Control | 31.8% | 68.2% | 100.0% |
|  | 43-52 years | Case-Control | Control | Count | 10 | 23 | 33 |
|  |  |  |  | % within Case-Control | 30.3% | 69.7% | 100.0% |
|  |  |  | Case | Count | 16 | 27 | 43 |
|  |  |  |  | % within Case-Control | 37.2% | 62.8% | 100.0% |
|  |  | Total | | Count | 26 | 50 | 76 |
|  |  |  |  | % within Case-Control | 34.2% | 65.8% | 100.0% |
|  | > 52 years | Case-Control | Control | Count | 5 | 28 | 33 |
|  |  |  |  | % within Case-Control | 15.2% | 84.8% | 100.0% |
|  |  |  | Case | Count | 19 | 27 | 46 |
|  |  |  |  | % within Case-Control | 41.3% | 58.7% | 100.0% |
|  |  | Total | | Count | 24 | 55 | 79 |
|  |  |  |  | % within Case-Control | 30.4% | 69.6% | 100.0% |
|  | Total | Case-Control | Control | Count | 44 | 116 | 160 |
|  |  |  |  | % within Case-Control | 27.5% | 72.5% | 100.0% |
|  |  |  | Case | Count | 63 | 97 | 160 |
|  |  |  |  | % within Case-Control | 39.4% | 60.6% | 100.0% |
|  |  | Total | | Count | 107 | 213 | 320 |
|  |  |  |  | % within Case-Control | 33.4% | 66.6% | 100.0% |
| 4 | < 32 years | Case-Control | Control | Count | 14 | 29 | 43 |
|  |  |  |  | % within Case-Control | 32.6% | 67.4% | 100.0% |
|  |  |  | Case | Count | 16 | 18 | 34 |
|  |  |  |  | % within Case-Control | 47.1% | 52.9% | 100.0% |
|  |  | Total | | Count | 30 | 47 | 77 |
|  |  |  |  | % within Case-Control | 39.0% | 61.0% | 100.0% |
|  | 32-42 years | Case-Control | Control | Count | 14 | 37 | 51 |
|  |  |  |  | % within Case-Control | 27.5% | 72.5% | 100.0% |
|  |  |  | Case | Count | 14 | 23 | 37 |
|  |  |  |  | % within Case-Control | 37.8% | 62.2% | 100.0% |
|  |  | Total | | Count | 28 | 60 | 88 |
|  |  |  |  | % within Case-Control | 31.8% | 68.2% | 100.0% |
|  | 43-52 years | Case-Control | Control | Count | 9 | 24 | 33 |
|  |  |  |  | % within Case-Control | 27.3% | 72.7% | 100.0% |
|  |  |  | Case | Count | 17 | 28 | 45 |
|  |  |  |  | % within Case-Control | 37.8% | 62.2% | 100.0% |
|  |  | Total | | Count | 26 | 52 | 78 |
|  |  |  |  | % within Case-Control | 33.3% | 66.7% | 100.0% |
|  | > 52 years | Case-Control | Control | Count | 7 | 26 | 33 |
|  |  |  |  | % within Case-Control | 21.2% | 78.8% | 100.0% |
|  |  |  | Case | Count | 16 | 28 | 44 |
|  |  |  |  | % within Case-Control | 36.4% | 63.6% | 100.0% |
|  |  | Total | | Count | 23 | 54 | 77 |
|  |  |  |  | % within Case-Control | 29.9% | 70.1% | 100.0% |
|  | Total | Case-Control | Control | Count | 44 | 116 | 160 |
|  |  |  |  | % within Case-Control | 27.5% | 72.5% | 100.0% |
|  |  |  | Case | Count | 63 | 97 | 160 |
|  |  |  |  | % within Case-Control | 39.4% | 60.6% | 100.0% |
|  |  | Total | | Count | 107 | 213 | 320 |
|  |  |  |  | % within Case-Control | 33.4% | 66.6% | 100.0% |
| 5 | < 32 years | Case-Control | Control | Count | 16 | 27 | 43 |
|  |  |  |  | % within Case-Control | 37.2% | 62.8% | 100.0% |
|  |  |  | Case | Count | 16 | 18 | 34 |
|  |  |  |  | % within Case-Control | 47.1% | 52.9% | 100.0% |
|  |  | Total | | Count | 32 | 45 | 77 |
|  |  |  |  | % within Case-Control | 41.6% | 58.4% | 100.0% |
|  | 32-42 years | Case-Control | Control | Count | 14 | 37 | 51 |
|  |  |  |  | % within Case-Control | 27.5% | 72.5% | 100.0% |
|  |  |  | Case | Count | 14 | 23 | 37 |
|  |  |  |  | % within Case-Control | 37.8% | 62.2% | 100.0% |
|  |  | Total | | Count | 28 | 60 | 88 |
|  |  |  |  | % within Case-Control | 31.8% | 68.2% | 100.0% |
|  | 43-52 years | Case-Control | Control | Count | 8 | 25 | 33 |
|  |  |  |  | % within Case-Control | 24.2% | 75.8% | 100.0% |
|  |  |  | Case | Count | 12 | 33 | 45 |
|  |  |  |  | % within Case-Control | 26.7% | 73.3% | 100.0% |
|  |  | Total | | Count | 20 | 58 | 78 |
|  |  |  |  | % within Case-Control | 25.6% | 74.4% | 100.0% |
|  | > 52 years | Case-Control | Control | Count | 7 | 26 | 33 |
|  |  |  |  | % within Case-Control | 21.2% | 78.8% | 100.0% |
|  |  |  | Case | Count | 20 | 24 | 44 |
|  |  |  |  | % within Case-Control | 45.5% | 54.5% | 100.0% |
|  |  | Total | | Count | 27 | 50 | 77 |
|  |  |  |  | % within Case-Control | 35.1% | 64.9% | 100.0% |
|  | Total | Case-Control | Control | Count | 45 | 115 | 160 |
|  |  |  |  | % within Case-Control | 28.1% | 71.9% | 100.0% |
|  |  |  | Case | Count | 62 | 98 | 160 |
|  |  |  |  | % within Case-Control | 38.8% | 61.3% | 100.0% |
|  |  | Total | | Count | 107 | 213 | 320 |
|  |  |  |  | % within Case-Control | 33.4% | 66.6% | 100.0% |
| Pooled | < 32 years | Case-Control | Control | Count | 15 | 28 | 43 |
|  |  |  | Case | Count | 15.2 | 19.4 | 34.6 |
|  |  | Total | | Count | 30.2 | 47.4 | 77.6 |
|  | 32-42 years | Case-Control | Control | Count | 14.4 | 36.6 | 51 |
|  |  |  | Case | Count | 13.4 | 23.4 | 36.8 |
|  |  | Total | | Count | 27.8 | 60 | 87.8 |
|  | 43-52 years | Case-Control | Control | Count | 8.2 | 24.8 | 33 |
|  |  |  | Case | Count | 15.4 | 28.6 | 44 |
|  |  | Total | | Count | 23.6 | 53.4 | 77 |
|  | > 52 years | Case-Control | Control | Count | 6.2 | 26.8 | 33 |
|  |  |  | Case | Count | 19 | 25.6 | 44.6 |
|  |  | Total | | Count | 25.2 | 52.4 | 77.6 |
|  | Total | Case-Control | Control | Count | 43.8 | 116.2 | 160 |
|  |  |  | Case | Count | 63 | 97 | 160 |
|  |  | Total | | Count | 106.8 | 213.2 | 320 |

**Chi-Square Test Case-Control and Income break down by Age (using pooled frequency)**

| **Case-Control * Income (dichotom) * Age Cross-tabulation** | | | | | | |
| --- | --- | --- | --- | --- | --- | --- |
| Age | | | | Income (dichotom) | | Total |
|  |  |  |  | High Income | Lower Middle Income |  |
| < 32 years | Case-Control | Control | Count | 15.0 | 28.0 | 43.0 |
|  |  |  | % within Case-Control | 34.9% | 65.1% | 100.0% |
|  |  | Case | Count | 15.2 | 19.4 | 34.6 |
|  |  |  | % within Case-Control | 43.9% | 56.1% | 100.0% |
|  | Total | | Count | 30.2 | 47.4 | 77.6 |
|  |  |  | % within Case-Control | 38.9% | 61.1% | 100.0% |
| 32-42 years | Case-Control | Control | Count | 14.4 | 36.6 | 51.0 |
|  |  |  | % within Case-Control | 28.2% | 71.8% | 100.0% |
|  |  | Case | Count | 13.4 | 23.4 | 36.8 |
|  |  |  | % within Case-Control | 36.4% | 63.6% | 100.0% |
|  | Total | | Count | 27.8 | 60.0 | 87.8 |
|  |  |  | % within Case-Control | 31.7% | 68.3% | 100.0% |
| 43-52 years | Case-Control | Control | Count | 8.2 | 24.8 | 33.0 |
|  |  |  | % within Case-Control | 24.8% | 75.2% | 100.0% |
|  |  | Case | Count | 15.4 | 28.6 | 44.0 |
|  |  |  | % within Case-Control | 35.0% | 65.0% | 100.0% |
|  | Total | | Count | 23.6 | 53.4 | 77.0 |
|  |  |  | % within Case-Control | 30.6% | 69.4% | 100.0% |
| > 52 years | Case-Control | Control | Count | 6.2 | 26.8 | 33.0 |
|  |  |  | % within Case-Control | 18.8% | 81.2% | 100.0% |
|  |  | Case | Count | 19.0 | 25.6 | 44.6 |
|  |  |  | % within Case-Control | 42.6% | 57.4% | 100.0% |
|  | Total | | Count | 25.2 | 52.4 | 77.6 |
|  |  |  | % within Case-Control | 32.5% | 67.5% | 100.0% |
| Total | Case-Control | Control | Count | 43.8 | 116.2 | 160.0 |
|  |  |  | % within Case-Control | 27.4% | 72.6% | 100.0% |
|  |  | Case | Count | 63.0 | 97.0 | 160.0 |
|  |  |  | % within Case-Control | 39.4% | 60.6% | 100.0% |
|  | Total | | Count | 106.8 | 213.2 | 320.0 |
|  |  |  | % within Case-Control | 33.4% | 66.6% | 100.0% |

| **Chi-Square Tests** | | | | | | |
| --- | --- | --- | --- | --- | --- | --- |
| Age | | Value | df | Asymptotic Significance (2-sided) | Exact Sig. (2-sided) | Exact Sig. (1-sided) |
| < 32 years | Pearson Chi-Square | .660^c^ | 1 | .417 |  |  |
|  | Continuity Correction^b^ | .334 | 1 | .563 |  |  |
|  | Likelihood Ratio | .659 | 1 | .417 |  |  |
|  | Fisher's Exact Test |  |  |  | .483 | .277 |
|  | Linear-by-Linear Association | .652 | 1 | .420 |  |  |
|  | N of Valid Cases | 78 |  |  |  |  |
| 32-42 years | Pearson Chi-Square | .661^d^ | 1 | .416 |  |  |
|  | Continuity Correction^b^ | .337 | 1 | .562 |  |  |
|  | Likelihood Ratio | .657 | 1 | .418 |  |  |
|  | Fisher's Exact Test |  |  |  | .482 | .265 |
|  | Linear-by-Linear Association | .653 | 1 | .419 |  |  |
|  | N of Valid Cases | 88 |  |  |  |  |
| 43-52 years | Pearson Chi-Square | .914^e^ | 1 | .339 |  |  |
|  | Continuity Correction^b^ | .499 | 1 | .480 |  |  |
|  | Likelihood Ratio | .926 | 1 | .336 |  |  |
|  | Fisher's Exact Test |  |  |  | .452 | .248 |
|  | Linear-by-Linear Association | .902 | 1 | .342 |  |  |
|  | N of Valid Cases | 77 |  |  |  |  |
| > 52 years | Pearson Chi-Square | 4.905^f^ | 1 | .027 |  |  |
|  | Continuity Correction^b^ | 3.879 | 1 | .049 |  |  |
|  | Likelihood Ratio | 5.102 | 1 | .024 |  |  |
|  | Fisher's Exact Test |  |  |  | .029 | .021 |
|  | Linear-by-Linear Association | 4.841 | 1 | .028 |  |  |
|  | N of Valid Cases | 78 |  |  |  |  |
| Total | Pearson Chi-Square | 5.181^a^ | 1 | .023 |  |  |
|  | Continuity Correction^b^ | 4.655 | 1 | .031 |  |  |
|  | Likelihood Ratio | 5.202 | 1 | .023 |  |  |
|  | Fisher's Exact Test |  |  |  | .033 | .016 |
|  | Linear-by-Linear Association | 5.165 | 1 | .023 |  |  |
|  | N of Valid Cases | 320 |  |  |  |  |
| a. 0 cells (0.0%) have expected count less than 5. The minimum expected count is 53.40. | | | | | | |
| b. Computed only for a 2x2 table | | | | | | |
| c. 0 cells (0.0%) have expected count less than 5. The minimum expected count is 13.47. | | | | | | |
| d. 0 cells (0.0%) have expected count less than 5. The minimum expected count is 11.65. | | | | | | |
| e. 0 cells (0.0%) have expected count less than 5. The minimum expected count is 10.11. | | | | | | |
| f. 0 cells (0.0%) have expected count less than 5. The minimum expected count is 10.72. | | | | | | |

1. **Cross-tabulation of Case-Control and Income break down by Education**

| **Case-Control * Income (dichotom) * Education Cross-tabulation** | | | | | | | |
| --- | --- | --- | --- | --- | --- | --- | --- |
| Imputation Number | Education | | | | Income (dichotom) | | Total |
|  |  |  |  |  | High Income | Lower Middle Income |  |
| Original data | High Education | Case-Control | Control | Count | 31 | 32 | 63 |
|  |  |  |  | % within Case-Control | 49.2% | 50.8% | 100.0% |
|  |  |  | Case | Count | 24 | 17 | 41 |
|  |  |  |  | % within Case-Control | 58.5% | 41.5% | 100.0% |
|  |  | Total | | Count | 55 | 49 | 104 |
|  |  |  |  | % within Case-Control | 52.9% | 47.1% | 100.0% |
|  | Middle Education | Case-Control | Control | Count | 2 | 44 | 46 |
|  |  |  |  | % within Case-Control | 4.3% | 95.7% | 100.0% |
|  |  |  | Case | Count | 8 | 27 | 35 |
|  |  |  |  | % within Case-Control | 22.9% | 77.1% | 100.0% |
|  |  | Total | | Count | 10 | 71 | 81 |
|  |  |  |  | % within Case-Control | 12.3% | 87.7% | 100.0% |
|  | Low Education | Case-Control | Control | Count | 0 | 23 | 23 |
|  |  |  |  | % within Case-Control | 0.0% | 100.0% | 100.0% |
|  |  |  | Case | Count | 2 | 10 | 12 |
|  |  |  |  | % within Case-Control | 16.7% | 83.3% | 100.0% |
|  |  | Total | | Count | 2 | 33 | 35 |
|  |  |  |  | % within Case-Control | 5.7% | 94.3% | 100.0% |
|  | Total | Case-Control | Control | Count | 33 | 99 | 132 |
|  |  |  |  | % within Case-Control | 25.0% | 75.0% | 100.0% |
|  |  |  | Case | Count | 34 | 54 | 88 |
|  |  |  |  | % within Case-Control | 38.6% | 61.4% | 100.0% |
|  |  | Total | | Count | 67 | 153 | 220 |
|  |  |  |  | % within Case-Control | 30.5% | 69.5% | 100.0% |
| 1 | High Education | Case-Control | Control | Count | 34 | 35 | 69 |
|  |  |  |  | % within Case-Control | 49.3% | 50.7% | 100.0% |
|  |  |  | Case | Count | 35 | 30 | 65 |
|  |  |  |  | % within Case-Control | 53.8% | 46.2% | 100.0% |
|  |  | Total | | Count | 69 | 65 | 134 |
|  |  |  |  | % within Case-Control | 51.5% | 48.5% | 100.0% |
|  | Middle Education | Case-Control | Control | Count | 8 | 53 | 61 |
|  |  |  |  | % within Case-Control | 13.1% | 86.9% | 100.0% |
|  |  |  | Case | Count | 20 | 55 | 75 |
|  |  |  |  | % within Case-Control | 26.7% | 73.3% | 100.0% |
|  |  | Total | | Count | 28 | 108 | 136 |
|  |  |  |  | % within Case-Control | 20.6% | 79.4% | 100.0% |
|  | Low Education | Case-Control | Control | Count | 3 | 27 | 30 |
|  |  |  |  | % within Case-Control | 10.0% | 90.0% | 100.0% |
|  |  |  | Case | Count | 6 | 14 | 20 |
|  |  |  |  | % within Case-Control | 30.0% | 70.0% | 100.0% |
|  |  | Total | | Count | 9 | 41 | 50 |
|  |  |  |  | % within Case-Control | 18.0% | 82.0% | 100.0% |
|  | Total | Case-Control | Control | Count | 45 | 115 | 160 |
|  |  |  |  | % within Case-Control | 28.1% | 71.9% | 100.0% |
|  |  |  | Case | Count | 61 | 99 | 160 |
|  |  |  |  | % within Case-Control | 38.1% | 61.9% | 100.0% |
|  |  | Total | | Count | 106 | 214 | 320 |
|  |  |  |  | % within Case-Control | 33.1% | 66.9% | 100.0% |
| 2 | High Education | Case-Control | Control | Count | 33 | 36 | 69 |
|  |  |  |  | % within Case-Control | 47.8% | 52.2% | 100.0% |
|  |  |  | Case | Count | 36 | 29 | 65 |
|  |  |  |  | % within Case-Control | 55.4% | 44.6% | 100.0% |
|  |  | Total | | Count | 69 | 65 | 134 |
|  |  |  |  | % within Case-Control | 51.5% | 48.5% | 100.0% |
|  | Middle Education | Case-Control | Control | Count | 5 | 56 | 61 |
|  |  |  |  | % within Case-Control | 8.2% | 91.8% | 100.0% |
|  |  |  | Case | Count | 24 | 51 | 75 |
|  |  |  |  | % within Case-Control | 32.0% | 68.0% | 100.0% |
|  |  | Total | | Count | 29 | 107 | 136 |
|  |  |  |  | % within Case-Control | 21.3% | 78.7% | 100.0% |
|  | Low Education | Case-Control | Control | Count | 3 | 27 | 30 |
|  |  |  |  | % within Case-Control | 10.0% | 90.0% | 100.0% |
|  |  |  | Case | Count | 6 | 14 | 20 |
|  |  |  |  | % within Case-Control | 30.0% | 70.0% | 100.0% |
|  |  | Total | | Count | 9 | 41 | 50 |
|  |  |  |  | % within Case-Control | 18.0% | 82.0% | 100.0% |
|  | Total | Case-Control | Control | Count | 41 | 119 | 160 |
|  |  |  |  | % within Case-Control | 25.6% | 74.4% | 100.0% |
|  |  |  | Case | Count | 66 | 94 | 160 |
|  |  |  |  | % within Case-Control | 41.3% | 58.8% | 100.0% |
|  |  | Total | | Count | 107 | 213 | 320 |
|  |  |  |  | % within Case-Control | 33.4% | 66.6% | 100.0% |
| 3 | High Education | Case-Control | Control | Count | 34 | 35 | 69 |
|  |  |  |  | % within Case-Control | 49.3% | 50.7% | 100.0% |
|  |  |  | Case | Count | 33 | 32 | 65 |
|  |  |  |  | % within Case-Control | 50.8% | 49.2% | 100.0% |
|  |  | Total | | Count | 67 | 67 | 134 |
|  |  |  |  | % within Case-Control | 50.0% | 50.0% | 100.0% |
|  | Middle Education | Case-Control | Control | Count | 7 | 54 | 61 |
|  |  |  |  | % within Case-Control | 11.5% | 88.5% | 100.0% |
|  |  |  | Case | Count | 23 | 52 | 75 |
|  |  |  |  | % within Case-Control | 30.7% | 69.3% | 100.0% |
|  |  | Total | | Count | 30 | 106 | 136 |
|  |  |  |  | % within Case-Control | 22.1% | 77.9% | 100.0% |
|  | Low Education | Case-Control | Control | Count | 3 | 27 | 30 |
|  |  |  |  | % within Case-Control | 10.0% | 90.0% | 100.0% |
|  |  |  | Case | Count | 7 | 13 | 20 |
|  |  |  |  | % within Case-Control | 35.0% | 65.0% | 100.0% |
|  |  | Total | | Count | 10 | 40 | 50 |
|  |  |  |  | % within Case-Control | 20.0% | 80.0% | 100.0% |
|  | Total | Case-Control | Control | Count | 44 | 116 | 160 |
|  |  |  |  | % within Case-Control | 27.5% | 72.5% | 100.0% |
|  |  |  | Case | Count | 63 | 97 | 160 |
|  |  |  |  | % within Case-Control | 39.4% | 60.6% | 100.0% |
|  |  | Total | | Count | 107 | 213 | 320 |
|  |  |  |  | % within Case-Control | 33.4% | 66.6% | 100.0% |
| 4 | High Education | Case-Control | Control | Count | 34 | 35 | 69 |
|  |  |  |  | % within Case-Control | 49.3% | 50.7% | 100.0% |
|  |  |  | Case | Count | 37 | 27 | 64 |
|  |  |  |  | % within Case-Control | 57.8% | 42.2% | 100.0% |
|  |  | Total | | Count | 71 | 62 | 133 |
|  |  |  |  | % within Case-Control | 53.4% | 46.6% | 100.0% |
|  | Middle Education | Case-Control | Control | Count | 7 | 54 | 61 |
|  |  |  |  | % within Case-Control | 11.5% | 88.5% | 100.0% |
|  |  |  | Case | Count | 22 | 54 | 76 |
|  |  |  |  | % within Case-Control | 28.9% | 71.1% | 100.0% |
|  |  | Total | | Count | 29 | 108 | 137 |
|  |  |  |  | % within Case-Control | 21.2% | 78.8% | 100.0% |
|  | Low Education | Case-Control | Control | Count | 3 | 27 | 30 |
|  |  |  |  | % within Case-Control | 10.0% | 90.0% | 100.0% |
|  |  |  | Case | Count | 4 | 16 | 20 |
|  |  |  |  | % within Case-Control | 20.0% | 80.0% | 100.0% |
|  |  | Total | | Count | 7 | 43 | 50 |
|  |  |  |  | % within Case-Control | 14.0% | 86.0% | 100.0% |
|  | Total | Case-Control | Control | Count | 44 | 116 | 160 |
|  |  |  |  | % within Case-Control | 27.5% | 72.5% | 100.0% |
|  |  |  | Case | Count | 63 | 97 | 160 |
|  |  |  |  | % within Case-Control | 39.4% | 60.6% | 100.0% |
|  |  | Total | | Count | 107 | 213 | 320 |
|  |  |  |  | % within Case-Control | 33.4% | 66.6% | 100.0% |
| 5 | High Education | Case-Control | Control | Count | 37 | 32 | 69 |
|  |  |  |  | % within Case-Control | 53.6% | 46.4% | 100.0% |
|  |  |  | Case | Count | 35 | 30 | 65 |
|  |  |  |  | % within Case-Control | 53.8% | 46.2% | 100.0% |
|  |  | Total | | Count | 72 | 62 | 134 |
|  |  |  |  | % within Case-Control | 53.7% | 46.3% | 100.0% |
|  | Middle Education | Case-Control | Control | Count | 6 | 55 | 61 |
|  |  |  |  | % within Case-Control | 9.8% | 90.2% | 100.0% |
|  |  |  | Case | Count | 22 | 53 | 75 |
|  |  |  |  | % within Case-Control | 29.3% | 70.7% | 100.0% |
|  |  | Total | | Count | 28 | 108 | 136 |
|  |  |  |  | % within Case-Control | 20.6% | 79.4% | 100.0% |
|  | Low Education | Case-Control | Control | Count | 2 | 28 | 30 |
|  |  |  |  | % within Case-Control | 6.7% | 93.3% | 100.0% |
|  |  |  | Case | Count | 5 | 15 | 20 |
|  |  |  |  | % within Case-Control | 25.0% | 75.0% | 100.0% |
|  |  | Total | | Count | 7 | 43 | 50 |
|  |  |  |  | % within Case-Control | 14.0% | 86.0% | 100.0% |
|  | Total | Case-Control | Control | Count | 45 | 115 | 160 |
|  |  |  |  | % within Case-Control | 28.1% | 71.9% | 100.0% |
|  |  |  | Case | Count | 62 | 98 | 160 |
|  |  |  |  | % within Case-Control | 38.8% | 61.3% | 100.0% |
|  |  | Total | | Count | 107 | 213 | 320 |
|  |  |  |  | % within Case-Control | 33.4% | 66.6% | 100.0% |
| Pooled | High Education | Case-Control | Control | Count | 34.4 | 34.6 | 69 |
|  |  |  | Case | Count | 35.2 | 29.6 | 64.8 |
|  |  | Total | | Count | 69.6 | 64.2 | 133.8 |
|  | Middle Education | Case-Control | Control | Count | 6.6 | 54.4 | 61 |
|  |  |  | Case | Count | 22.2 | 53 | 75.2 |
|  |  | Total | | Count | 28.8 | 107.4 | 136.2 |
|  | Low Education | Case-Control | Control | Count | 2.8 | 27.2 | 30 |
|  |  |  | Case | Count | 5.6 | 14.4 | 20 |
|  |  | Total | | Count | 8.4 | 41.6 | 50 |
|  | Total | Case-Control | Control | Count | 43.8 | 116.2 | 160 |
|  |  |  | Case | Count | 63 | 97 | 160 |
|  |  | Total | | Count | 106.8 | 213.2 | 320 |

**Chi-Square Test Case-Control and Income break down by Education (using pooled frequency)**

| **Case-Control * Income (dichotom) * Education Cross-tabulation** | | | | | | |
| --- | --- | --- | --- | --- | --- | --- |
| Education | | | | Income (dichotom) | | Total |
|  |  |  |  | High Income | Lower Middle Income |  |
| High Education | Case-Control | Control | Count | 34.4 | 34.6 | 69.0 |
|  |  |  | % within Case-Control | 49.9% | 50.1% | 100.0% |
|  |  | Case | Count | 35.2 | 29.6 | 64.8 |
|  |  |  | % within Case-Control | 54.3% | 45.7% | 100.0% |
|  | Total | | Count | 69.6 | 64.2 | 133.8 |
|  |  |  | % within Case-Control | 52.0% | 48.0% | 100.0% |
| Middle Education | Case-Control | Control | Count | 6.6 | 54.4 | 61.0 |
|  |  |  | % within Case-Control | 10.8% | 89.2% | 100.0% |
|  |  | Case | Count | 22.2 | 53.0 | 75.2 |
|  |  |  | % within Case-Control | 29.5% | 70.5% | 100.0% |
|  | Total | | Count | 28.8 | 107.4 | 136.2 |
|  |  |  | % within Case-Control | 21.1% | 78.9% | 100.0% |
| Low Education | Case-Control | Control | Count | 2.8 | 27.2 | 30.0 |
|  |  |  | % within Case-Control | 9.3% | 90.7% | 100.0% |
|  |  | Case | Count | 5.6 | 14.4 | 20.0 |
|  |  |  | % within Case-Control | 28.0% | 72.0% | 100.0% |
|  | Total | | Count | 8.4 | 41.6 | 50.0 |
|  |  |  | % within Case-Control | 16.8% | 83.2% | 100.0% |
| Total | Case-Control | Control | Count | 43.8 | 116.2 | 160.0 |
|  |  |  | % within Case-Control | 27.4% | 72.6% | 100.0% |
|  |  | Case | Count | 63.0 | 97.0 | 160.0 |
|  |  |  | % within Case-Control | 39.4% | 60.6% | 100.0% |
|  | Total | | Count | 106.8 | 213.2 | 320.0 |
|  |  |  | % within Case-Control | 33.4% | 66.6% | 100.0% |

| **Chi-Square Tests** | | | | | | |
| --- | --- | --- | --- | --- | --- | --- |
| Education | | Value | df | Asymptotic Significance (2-sided) | Exact Sig. (2-sided) | Exact Sig. (1-sided) |
| High Education | Pearson Chi-Square | .267^c^ | 1 | .605 |  |  |
|  | Continuity Correction^b^ | .118 | 1 | .731 |  |  |
|  | Likelihood Ratio | .267 | 1 | .605 |  |  |
|  | Fisher's Exact Test |  |  |  | .609 | .361 |
|  | Linear-by-Linear Association | .265 | 1 | .607 |  |  |
|  | N of Valid Cases | 134 |  |  |  |  |
| Middle Education | Pearson Chi-Square | 7.065^d^ | 1 | .008 |  |  |
|  | Continuity Correction^b^ | 5.987 | 1 | .014 |  |  |
|  | Likelihood Ratio | 7.456 | 1 | .006 |  |  |
|  | Fisher's Exact Test |  |  |  | .012 | .009 |
|  | Linear-by-Linear Association | 7.013 | 1 | .008 |  |  |
|  | N of Valid Cases | 136 |  |  |  |  |
| Low Education | Pearson Chi-Square | 2.991^e^ | 1 | .084 |  |  |
|  | Continuity Correction^b^ | 1.805 | 1 | .179 |  |  |
|  | Likelihood Ratio | 2.941 | 1 | .086 |  |  |
|  | Fisher's Exact Test |  |  |  | .130 | .078 |
|  | Linear-by-Linear Association | 2.932 | 1 | .087 |  |  |
|  | N of Valid Cases | 50 |  |  |  |  |
| Total | Pearson Chi-Square | 5.181^a^ | 1 | .023 |  |  |
|  | Continuity Correction^b^ | 4.655 | 1 | .031 |  |  |
|  | Likelihood Ratio | 5.202 | 1 | .023 |  |  |
|  | Fisher's Exact Test |  |  |  | .033 | .016 |
|  | Linear-by-Linear Association | 5.165 | 1 | .023 |  |  |
|  | N of Valid Cases | 320 |  |  |  |  |
| a. 0 cells (0.0%) have expected count less than 5. The minimum expected count is 53.40. | | | | | | |
| b. Computed only for a 2x2 table | | | | | | |
| c. 0 cells (0.0%) have expected count less than 5. The minimum expected count is 31.09. | | | | | | |
| d. 0 cells (0.0%) have expected count less than 5. The minimum expected count is 12.90. | | | | | | |
| e. 1 cells (25.0%) have expected count less than 5. The minimum expected count is 3.36. | | | | | | |

1. **Cross-tabulation of Case-Control and Income break down by Occupation**

| **Case-Control * Income (dichotom) * Occupation Cross-tabulation** | | | | | | | |
| --- | --- | --- | --- | --- | --- | --- | --- |
| Imputation Number | Occupation | | | | Income (dichotom) | | Total |
|  |  |  |  |  | High Income | Lower Middle Income |  |
| Original data | Unemployed | Case-Control | Control | Count |  | 6 | 6 |
|  |  |  |  | % within Case-Control |  | 100.0% | 100.0% |
|  |  |  | Case | Count |  | 9 | 9 |
|  |  |  |  | % within Case-Control |  | 100.0% | 100.0% |
|  |  | Total | | Count |  | 15 | 15 |
|  |  |  |  | % within Case-Control |  | 100.0% | 100.0% |
|  | Housewife | Case-Control | Control | Count |  | 35 | 35 |
|  |  |  |  | % within Case-Control |  | 100.0% | 100.0% |
|  |  |  | Case | Count |  | 13 | 13 |
|  |  |  |  | % within Case-Control |  | 100.0% | 100.0% |
|  |  | Total | | Count |  | 48 | 48 |
|  |  |  |  | % within Case-Control |  | 100.0% | 100.0% |
|  | Retired | Case-Control | Control | Count | 3 | 0 | 3 |
|  |  |  |  | % within Case-Control | 100.0% | 0.0% | 100.0% |
|  |  |  | Case | Count | 6 | 2 | 8 |
|  |  |  |  | % within Case-Control | 75.0% | 25.0% | 100.0% |
|  |  | Total | | Count | 9 | 2 | 11 |
|  |  |  |  | % within Case-Control | 81.8% | 18.2% | 100.0% |
|  | Civil Servant | Case-Control | Control | Count | 9 | 10 | 19 |
|  |  |  |  | % within Case-Control | 47.4% | 52.6% | 100.0% |
|  |  |  | Case | Count | 6 | 5 | 11 |
|  |  |  |  | % within Case-Control | 54.5% | 45.5% | 100.0% |
|  |  | Total | | Count | 15 | 15 | 30 |
|  |  |  |  | % within Case-Control | 50.0% | 50.0% | 100.0% |
|  | Private | Case-Control | Control | Count | 21 | 47 | 68 |
|  |  |  |  | % within Case-Control | 30.9% | 69.1% | 100.0% |
|  |  |  | Case | Count | 19 | 22 | 41 |
|  |  |  |  | % within Case-Control | 46.3% | 53.7% | 100.0% |
|  |  | Total | | Count | 40 | 69 | 109 |
|  |  |  |  | % within Case-Control | 36.7% | 63.3% | 100.0% |
|  | Total | Case-Control | Control | Count | 33 | 98 | 131 |
|  |  |  |  | % within Case-Control | 25.2% | 74.8% | 100.0% |
|  |  |  | Case | Count | 31 | 51 | 82 |
|  |  |  |  | % within Case-Control | 37.8% | 62.2% | 100.0% |
|  |  | Total | | Count | 64 | 149 | 213 |
|  |  |  |  | % within Case-Control | 30.0% | 70.0% | 100.0% |
| 1 | Unemployed | Case-Control | Control | Count | 1 | 7 | 8 |
|  |  |  |  | % within Case-Control | 12.5% | 87.5% | 100.0% |
|  |  |  | Case | Count | 0 | 12 | 12 |
|  |  |  |  | % within Case-Control | 0.0% | 100.0% | 100.0% |
|  |  | Total | | Count | 1 | 19 | 20 |
|  |  |  |  | % within Case-Control | 5.0% | 95.0% | 100.0% |
|  | Housewife | Case-Control | Control | Count | 3 | 45 | 48 |
|  |  |  |  | % within Case-Control | 6.3% | 93.8% | 100.0% |
|  |  |  | Case | Count | 9 | 38 | 47 |
|  |  |  |  | % within Case-Control | 19.1% | 80.9% | 100.0% |
|  |  | Total | | Count | 12 | 83 | 95 |
|  |  |  |  | % within Case-Control | 12.6% | 87.4% | 100.0% |
|  | Retired | Case-Control | Control | Count | 3 | 0 | 3 |
|  |  |  |  | % within Case-Control | 100.0% | 0.0% | 100.0% |
|  |  |  | Case | Count | 7 | 5 | 12 |
|  |  |  |  | % within Case-Control | 58.3% | 41.7% | 100.0% |
|  |  | Total | | Count | 10 | 5 | 15 |
|  |  |  |  | % within Case-Control | 66.7% | 33.3% | 100.0% |
|  | Civil Servant | Case-Control | Control | Count | 9 | 10 | 19 |
|  |  |  |  | % within Case-Control | 47.4% | 52.6% | 100.0% |
|  |  |  | Case | Count | 10 | 8 | 18 |
|  |  |  |  | % within Case-Control | 55.6% | 44.4% | 100.0% |
|  |  | Total | | Count | 19 | 18 | 37 |
|  |  |  |  | % within Case-Control | 51.4% | 48.6% | 100.0% |
|  | Private | Case-Control | Control | Count | 29 | 53 | 82 |
|  |  |  |  | % within Case-Control | 35.4% | 64.6% | 100.0% |
|  |  |  | Case | Count | 35 | 36 | 71 |
|  |  |  |  | % within Case-Control | 49.3% | 50.7% | 100.0% |
|  |  | Total | | Count | 64 | 89 | 153 |
|  |  |  |  | % within Case-Control | 41.8% | 58.2% | 100.0% |
|  | Total | Case-Control | Control | Count | 45 | 115 | 160 |
|  |  |  |  | % within Case-Control | 28.1% | 71.9% | 100.0% |
|  |  |  | Case | Count | 61 | 99 | 160 |
|  |  |  |  | % within Case-Control | 38.1% | 61.9% | 100.0% |
|  |  | Total | | Count | 106 | 214 | 320 |
|  |  |  |  | % within Case-Control | 33.1% | 66.9% | 100.0% |
| 2 | Unemployed | Case-Control | Control | Count | 0 | 7 | 7 |
|  |  |  |  | % within Case-Control | 0.0% | 100.0% | 100.0% |
|  |  |  | Case | Count | 1 | 12 | 13 |
|  |  |  |  | % within Case-Control | 7.7% | 92.3% | 100.0% |
|  |  | Total | | Count | 1 | 19 | 20 |
|  |  |  |  | % within Case-Control | 5.0% | 95.0% | 100.0% |
|  | Housewife | Case-Control | Control | Count | 2 | 47 | 49 |
|  |  |  |  | % within Case-Control | 4.1% | 95.9% | 100.0% |
|  |  |  | Case | Count | 11 | 37 | 48 |
|  |  |  |  | % within Case-Control | 22.9% | 77.1% | 100.0% |
|  |  | Total | | Count | 13 | 84 | 97 |
|  |  |  |  | % within Case-Control | 13.4% | 86.6% | 100.0% |
|  | Retired | Case-Control | Control | Count | 3 | 0 | 3 |
|  |  |  |  | % within Case-Control | 100.0% | 0.0% | 100.0% |
|  |  |  | Case | Count | 8 | 2 | 10 |
|  |  |  |  | % within Case-Control | 80.0% | 20.0% | 100.0% |
|  |  | Total | | Count | 11 | 2 | 13 |
|  |  |  |  | % within Case-Control | 84.6% | 15.4% | 100.0% |
|  | Civil Servant | Case-Control | Control | Count | 9 | 10 | 19 |
|  |  |  |  | % within Case-Control | 47.4% | 52.6% | 100.0% |
|  |  |  | Case | Count | 10 | 8 | 18 |
|  |  |  |  | % within Case-Control | 55.6% | 44.4% | 100.0% |
|  |  | Total | | Count | 19 | 18 | 37 |
|  |  |  |  | % within Case-Control | 51.4% | 48.6% | 100.0% |
|  | Private | Case-Control | Control | Count | 27 | 55 | 82 |
|  |  |  |  | % within Case-Control | 32.9% | 67.1% | 100.0% |
|  |  |  | Case | Count | 36 | 35 | 71 |
|  |  |  |  | % within Case-Control | 50.7% | 49.3% | 100.0% |
|  |  | Total | | Count | 63 | 90 | 153 |
|  |  |  |  | % within Case-Control | 41.2% | 58.8% | 100.0% |
|  | Total | Case-Control | Control | Count | 41 | 119 | 160 |
|  |  |  |  | % within Case-Control | 25.6% | 74.4% | 100.0% |
|  |  |  | Case | Count | 66 | 94 | 160 |
|  |  |  |  | % within Case-Control | 41.3% | 58.8% | 100.0% |
|  |  | Total | | Count | 107 | 213 | 320 |
|  |  |  |  | % within Case-Control | 33.4% | 66.6% | 100.0% |
| 3 | Unemployed | Case-Control | Control | Count | 0 | 7 | 7 |
|  |  |  |  | % within Case-Control | 0.0% | 100.0% | 100.0% |
|  |  |  | Case | Count | 1 | 11 | 12 |
|  |  |  |  | % within Case-Control | 8.3% | 91.7% | 100.0% |
|  |  | Total | | Count | 1 | 18 | 19 |
|  |  |  |  | % within Case-Control | 5.3% | 94.7% | 100.0% |
|  | Housewife | Case-Control | Control | Count | 4 | 45 | 49 |
|  |  |  |  | % within Case-Control | 8.2% | 91.8% | 100.0% |
|  |  |  | Case | Count | 11 | 36 | 47 |
|  |  |  |  | % within Case-Control | 23.4% | 76.6% | 100.0% |
|  |  | Total | | Count | 15 | 81 | 96 |
|  |  |  |  | % within Case-Control | 15.6% | 84.4% | 100.0% |
|  | Retired | Case-Control | Control | Count | 3 | 0 | 3 |
|  |  |  |  | % within Case-Control | 100.0% | 0.0% | 100.0% |
|  |  |  | Case | Count | 8 | 4 | 12 |
|  |  |  |  | % within Case-Control | 66.7% | 33.3% | 100.0% |
|  |  | Total | | Count | 11 | 4 | 15 |
|  |  |  |  | % within Case-Control | 73.3% | 26.7% | 100.0% |
|  | Civil Servant | Case-Control | Control | Count | 9 | 10 | 19 |
|  |  |  |  | % within Case-Control | 47.4% | 52.6% | 100.0% |
|  |  |  | Case | Count | 9 | 8 | 17 |
|  |  |  |  | % within Case-Control | 52.9% | 47.1% | 100.0% |
|  |  | Total | | Count | 18 | 18 | 36 |
|  |  |  |  | % within Case-Control | 50.0% | 50.0% | 100.0% |
|  | Private | Case-Control | Control | Count | 28 | 54 | 82 |
|  |  |  |  | % within Case-Control | 34.1% | 65.9% | 100.0% |
|  |  |  | Case | Count | 34 | 38 | 72 |
|  |  |  |  | % within Case-Control | 47.2% | 52.8% | 100.0% |
|  |  | Total | | Count | 62 | 92 | 154 |
|  |  |  |  | % within Case-Control | 40.3% | 59.7% | 100.0% |
|  | Total | Case-Control | Control | Count | 44 | 116 | 160 |
|  |  |  |  | % within Case-Control | 27.5% | 72.5% | 100.0% |
|  |  |  | Case | Count | 63 | 97 | 160 |
|  |  |  |  | % within Case-Control | 39.4% | 60.6% | 100.0% |
|  |  | Total | | Count | 107 | 213 | 320 |
|  |  |  |  | % within Case-Control | 33.4% | 66.6% | 100.0% |
| 4 | Unemployed | Case-Control | Control | Count |  | 7 | 7 |
|  |  |  |  | % within Case-Control |  | 100.0% | 100.0% |
|  |  |  | Case | Count |  | 14 | 14 |
|  |  |  |  | % within Case-Control |  | 100.0% | 100.0% |
|  |  | Total | | Count |  | 21 | 21 |
|  |  |  |  | % within Case-Control |  | 100.0% | 100.0% |
|  | Housewife | Case-Control | Control | Count | 5 | 44 | 49 |
|  |  |  |  | % within Case-Control | 10.2% | 89.8% | 100.0% |
|  |  |  | Case | Count | 7 | 39 | 46 |
|  |  |  |  | % within Case-Control | 15.2% | 84.8% | 100.0% |
|  |  | Total | | Count | 12 | 83 | 95 |
|  |  |  |  | % within Case-Control | 12.6% | 87.4% | 100.0% |
|  | Retired | Case-Control | Control | Count | 3 | 0 | 3 |
|  |  |  |  | % within Case-Control | 100.0% | 0.0% | 100.0% |
|  |  |  | Case | Count | 7 | 3 | 10 |
|  |  |  |  | % within Case-Control | 70.0% | 30.0% | 100.0% |
|  |  | Total | | Count | 10 | 3 | 13 |
|  |  |  |  | % within Case-Control | 76.9% | 23.1% | 100.0% |
|  | Civil Servant | Case-Control | Control | Count | 9 | 10 | 19 |
|  |  |  |  | % within Case-Control | 47.4% | 52.6% | 100.0% |
|  |  |  | Case | Count | 9 | 7 | 16 |
|  |  |  |  | % within Case-Control | 56.3% | 43.8% | 100.0% |
|  |  | Total | | Count | 18 | 17 | 35 |
|  |  |  |  | % within Case-Control | 51.4% | 48.6% | 100.0% |
|  | Private | Case-Control | Control | Count | 27 | 55 | 82 |
|  |  |  |  | % within Case-Control | 32.9% | 67.1% | 100.0% |
|  |  |  | Case | Count | 40 | 34 | 74 |
|  |  |  |  | % within Case-Control | 54.1% | 45.9% | 100.0% |
|  |  | Total | | Count | 67 | 89 | 156 |
|  |  |  |  | % within Case-Control | 42.9% | 57.1% | 100.0% |
|  | Total | Case-Control | Control | Count | 44 | 116 | 160 |
|  |  |  |  | % within Case-Control | 27.5% | 72.5% | 100.0% |
|  |  |  | Case | Count | 63 | 97 | 160 |
|  |  |  |  | % within Case-Control | 39.4% | 60.6% | 100.0% |
|  |  | Total | | Count | 107 | 213 | 320 |
|  |  |  |  | % within Case-Control | 33.4% | 66.6% | 100.0% |
| 5 | Unemployed | Case-Control | Control | Count | 0 | 8 | 8 |
|  |  |  |  | % within Case-Control | 0.0% | 100.0% | 100.0% |
|  |  |  | Case | Count | 1 | 11 | 12 |
|  |  |  |  | % within Case-Control | 8.3% | 91.7% | 100.0% |
|  |  | Total | | Count | 1 | 19 | 20 |
|  |  |  |  | % within Case-Control | 5.0% | 95.0% | 100.0% |
|  | Housewife | Case-Control | Control | Count | 4 | 44 | 48 |
|  |  |  |  | % within Case-Control | 8.3% | 91.7% | 100.0% |
|  |  |  | Case | Count | 6 | 40 | 46 |
|  |  |  |  | % within Case-Control | 13.0% | 87.0% | 100.0% |
|  |  | Total | | Count | 10 | 84 | 94 |
|  |  |  |  | % within Case-Control | 10.6% | 89.4% | 100.0% |
|  | Retired | Case-Control | Control | Count | 3 | 0 | 3 |
|  |  |  |  | % within Case-Control | 100.0% | 0.0% | 100.0% |
|  |  |  | Case | Count | 7 | 4 | 11 |
|  |  |  |  | % within Case-Control | 63.6% | 36.4% | 100.0% |
|  |  | Total | | Count | 10 | 4 | 14 |
|  |  |  |  | % within Case-Control | 71.4% | 28.6% | 100.0% |
|  | Civil Servant | Case-Control | Control | Count | 9 | 10 | 19 |
|  |  |  |  | % within Case-Control | 47.4% | 52.6% | 100.0% |
|  |  |  | Case | Count | 12 | 6 | 18 |
|  |  |  |  | % within Case-Control | 66.7% | 33.3% | 100.0% |
|  |  | Total | | Count | 21 | 16 | 37 |
|  |  |  |  | % within Case-Control | 56.8% | 43.2% | 100.0% |
|  | Private | Case-Control | Control | Count | 29 | 53 | 82 |
|  |  |  |  | % within Case-Control | 35.4% | 64.6% | 100.0% |
|  |  |  | Case | Count | 36 | 37 | 73 |
|  |  |  |  | % within Case-Control | 49.3% | 50.7% | 100.0% |
|  |  | Total | | Count | 65 | 90 | 155 |
|  |  |  |  | % within Case-Control | 41.9% | 58.1% | 100.0% |
|  | Total | Case-Control | Control | Count | 45 | 115 | 160 |
|  |  |  |  | % within Case-Control | 28.1% | 71.9% | 100.0% |
|  |  |  | Case | Count | 62 | 98 | 160 |
|  |  |  |  | % within Case-Control | 38.8% | 61.3% | 100.0% |
|  |  | Total | | Count | 107 | 213 | 320 |
|  |  |  |  | % within Case-Control | 33.4% | 66.6% | 100.0% |
| Pooled | Unemployed | Case-Control | Control | Count | .2 | 7.2 | 7.4 |
|  |  |  | Case | Count | .6 | 12 | 12.6 |
|  |  | Total | | Count | .8 | 19.2 | 20 |
|  | Housewife | Case-Control | Control | Count | 3.6 | 45 | 48.6 |
|  |  |  | Case | Count | 8.8 | 38 | 46.8 |
|  |  | Total | | Count | 12.4 | 83 | 95.4 |
|  | Retired | Case-Control | Control | Count | 3 | 0 | 3 |
|  |  |  | Case | Count | 7.4 | 3.6 | 11 |
|  |  | Total | | Count | 10.4 | 3.6 | 14 |
|  | Civil Servant | Case-Control | Control | Count | 9 | 10 | 19 |
|  |  |  | Case | Count | 10 | 7.4 | 17.4 |
|  |  | Total | | Count | 19 | 17.4 | 36.4 |
|  | Private | Case-Control | Control | Count | 28 | 54 | 82 |
|  |  |  | Case | Count | 36.2 | 36 | 72.2 |
|  |  | Total | | Count | 64.2 | 90 | 154.2 |
|  | Total | Case-Control | Control | Count | 43.8 | 116.2 | 160 |
|  |  |  | Case | Count | 63 | 97 | 160 |
|  |  | Total | | Count | 106.8 | 213.2 | 320 |

**Chi-Square Test Case-Control and Income break down by Occupation (using pooled frequency)**

| **Case-Control * Income (dichotom) * Occupation Cross-tabulation** | | | | | | |
| --- | --- | --- | --- | --- | --- | --- |
| Occupation | | | | Income (dichotom) | | Total |
|  |  |  |  | High Income | Lower Middle Income |  |
| Unemployed | Case-Control | Control | Count | .2 | 7.2 | 7.4 |
|  |  |  | % within Case-Control | 2.7% | 97.3% | 100.0% |
|  |  | Case | Count | .6 | 12.0 | 12.6 |
|  |  |  | % within Case-Control | 4.8% | 95.2% | 100.0% |
|  | Total | | Count | .8 | 19.2 | 20.0 |
|  |  |  | % within Case-Control | 4.0% | 96.0% | 100.0% |
| Housewife | Case-Control | Control | Count | 3.6 | 45.0 | 48.6 |
|  |  |  | % within Case-Control | 7.4% | 92.6% | 100.0% |
|  |  | Case | Count | 8.8 | 38.0 | 46.8 |
|  |  |  | % within Case-Control | 18.8% | 81.2% | 100.0% |
|  | Total | | Count | 12.4 | 83.0 | 95.4 |
|  |  |  | % within Case-Control | 13.0% | 87.0% | 100.0% |
| Retired | Case-Control | Control | Count | 3.0 | .0 | 3.0 |
|  |  |  | % within Case-Control | 100.0% | 0.0% | 100.0% |
|  |  | Case | Count | 7.4 | 3.6 | 11.0 |
|  |  |  | % within Case-Control | 67.3% | 32.7% | 100.0% |
|  | Total | | Count | 10.4 | 3.6 | 14.0 |
|  |  |  | % within Case-Control | 74.3% | 25.7% | 100.0% |
| Civil Servant | Case-Control | Control | Count | 9.0 | 10.0 | 19.0 |
|  |  |  | % within Case-Control | 47.4% | 52.6% | 100.0% |
|  |  | Case | Count | 10.0 | 7.4 | 17.4 |
|  |  |  | % within Case-Control | 57.5% | 42.5% | 100.0% |
|  | Total | | Count | 19.0 | 17.4 | 36.4 |
|  |  |  | % within Case-Control | 52.2% | 47.8% | 100.0% |
| Private | Case-Control | Control | Count | 28.0 | 54.0 | 82.0 |
|  |  |  | % within Case-Control | 34.1% | 65.9% | 100.0% |
|  |  | Case | Count | 36.2 | 36.0 | 72.2 |
|  |  |  | % within Case-Control | 50.1% | 49.9% | 100.0% |
|  | Total | | Count | 64.2 | 90.0 | 154.2 |
|  |  |  | % within Case-Control | 41.6% | 58.4% | 100.0% |
| Total | Case-Control | Control | Count | 43.8 | 116.2 | 160.0 |
|  |  |  | % within Case-Control | 27.4% | 72.6% | 100.0% |
|  |  | Case | Count | 63.0 | 97.0 | 160.0 |
|  |  |  | % within Case-Control | 39.4% | 60.6% | 100.0% |
|  | Total | | Count | 106.8 | 213.2 | 320.0 |
|  |  |  | % within Case-Control | 33.4% | 66.6% | 100.0% |

| **Chi-Square Tests** | | | | | | |
| --- | --- | --- | --- | --- | --- | --- |
| Occupation | | Value | df | Asymptotic Significance (2-sided) | Exact Sig. (2-sided) | Exact Sig. (1-sided) |
| Unemployed | Pearson Chi-Square | .051^c^ | 1 | .821 |  |  |
|  | Continuity Correction^b^ | .000 | 1 | 1.000 |  |  |
|  | Likelihood Ratio | .054 | 1 | .815 |  |  |
|  | Fisher's Exact Test |  |  |  | 1.000 | .650 |
|  | Linear-by-Linear Association | .049 | 1 | .825 |  |  |
|  | N of Valid Cases | 20 |  |  |  |  |
| Housewife | Pearson Chi-Square | 2.738^d^ | 1 | .098 |  |  |
|  | Continuity Correction^b^ | 1.823 | 1 | .177 |  |  |
|  | Likelihood Ratio | 2.807 | 1 | .094 |  |  |
|  | Fisher's Exact Test |  |  |  | .143 | .101 |
|  | Linear-by-Linear Association | 2.709 | 1 | .100 |  |  |
|  | N of Valid Cases | 95 |  |  |  |  |
| Retired | Pearson Chi-Square | 1.322^e^ | 1 | .250 |  |  |
|  | Continuity Correction^b^ | .164 | 1 | .686 |  |  |
|  | Likelihood Ratio | 2.052 | 1 | .152 |  |  |
|  | Fisher's Exact Test |  |  |  | .505 | .330 |
|  | Linear-by-Linear Association | 1.227 | 1 | .268 |  |  |
|  | N of Valid Cases | 14 |  |  |  |  |
| Civil Servant | Pearson Chi-Square | .372^f^ | 1 | .542 |  |  |
|  | Continuity Correction^b^ | .077 | 1 | .781 |  |  |
|  | Likelihood Ratio | .372 | 1 | .542 |  |  |
|  | Fisher's Exact Test |  |  |  | .525 | .363 |
|  | Linear-by-Linear Association | .361 | 1 | .548 |  |  |
|  | N of Valid Cases | 36 |  |  |  |  |
| Private | Pearson Chi-Square | 4.041^g^ | 1 | .044 |  |  |
|  | Continuity Correction^b^ | 3.410 | 1 | .065 |  |  |
|  | Likelihood Ratio | 4.051 | 1 | .044 |  |  |
|  | Fisher's Exact Test |  |  |  | .051 | .034 |
|  | Linear-by-Linear Association | 4.015 | 1 | .045 |  |  |
|  | N of Valid Cases | 154 |  |  |  |  |
| Total | Pearson Chi-Square | 5.181^a^ | 1 | .023 |  |  |
|  | Continuity Correction^b^ | 4.655 | 1 | .031 |  |  |
|  | Likelihood Ratio | 5.202 | 1 | .023 |  |  |
|  | Fisher's Exact Test |  |  |  | .033 | .016 |
|  | Linear-by-Linear Association | 5.165 | 1 | .023 |  |  |
|  | N of Valid Cases | 320 |  |  |  |  |
| a. 0 cells (0.0%) have expected count less than 5. The minimum expected count is 53.40. | | | | | | |
| b. Computed only for a 2x2 table | | | | | | |
| c. 2 cells (50.0%) have expected count less than 5. The minimum expected count is .30. | | | | | | |
| d. 0 cells (0.0%) have expected count less than 5. The minimum expected count is 6.08. | | | | | | |
| e. 3 cells (75.0%) have expected count less than 5. The minimum expected count is .77. | | | | | | |
| f. 0 cells (0.0%) have expected count less than 5. The minimum expected count is 8.32. | | | | | | |
| g. 0 cells (0.0%) have expected count less than 5. The minimum expected count is 30.06. | | | | | | |

1. **Cross-tabulation of Case-Control and Income break down by Hours Worked**

| **Case-Control * Income (dichotom) * Hours Worked Cross-tabulation** | | | | | | | |
| --- | --- | --- | --- | --- | --- | --- | --- |
| Imputation Number | Hours Worked | | | | Income (dichotom) | | Total |
|  |  |  |  |  | High Income | Lower Middle Income |  |
| Original data | < 6 hours | Case-Control | Control | Count | 7 | 44 | 51 |
|  |  |  |  | % within Case-Control | 13.7% | 86.3% | 100.0% |
|  |  |  | Case | Count | 10 | 26 | 36 |
|  |  |  |  | % within Case-Control | 27.8% | 72.2% | 100.0% |
|  |  | Total | | Count | 17 | 70 | 87 |
|  |  |  |  | % within Case-Control | 19.5% | 80.5% | 100.0% |
|  | >= 6 hours | Case-Control | Control | Count | 26 | 55 | 81 |
|  |  |  |  | % within Case-Control | 32.1% | 67.9% | 100.0% |
|  |  |  | Case | Count | 22 | 24 | 46 |
|  |  |  |  | % within Case-Control | 47.8% | 52.2% | 100.0% |
|  |  | Total | | Count | 48 | 79 | 127 |
|  |  |  |  | % within Case-Control | 37.8% | 62.2% | 100.0% |
|  | Total | Case-Control | Control | Count | 33 | 99 | 132 |
|  |  |  |  | % within Case-Control | 25.0% | 75.0% | 100.0% |
|  |  |  | Case | Count | 32 | 50 | 82 |
|  |  |  |  | % within Case-Control | 39.0% | 61.0% | 100.0% |
|  |  | Total | | Count | 65 | 149 | 214 |
|  |  |  |  | % within Case-Control | 30.4% | 69.6% | 100.0% |
| 1 | < 6 hours | Case-Control | Control | Count | 11 | 55 | 66 |
|  |  |  |  | % within Case-Control | 16.7% | 83.3% | 100.0% |
|  |  |  | Case | Count | 17 | 52 | 69 |
|  |  |  |  | % within Case-Control | 24.6% | 75.4% | 100.0% |
|  |  | Total | | Count | 28 | 107 | 135 |
|  |  |  |  | % within Case-Control | 20.7% | 79.3% | 100.0% |
|  | >= 6 hours | Case-Control | Control | Count | 34 | 60 | 94 |
|  |  |  |  | % within Case-Control | 36.2% | 63.8% | 100.0% |
|  |  |  | Case | Count | 44 | 47 | 91 |
|  |  |  |  | % within Case-Control | 48.4% | 51.6% | 100.0% |
|  |  | Total | | Count | 78 | 107 | 185 |
|  |  |  |  | % within Case-Control | 42.2% | 57.8% | 100.0% |
|  | Total | Case-Control | Control | Count | 45 | 115 | 160 |
|  |  |  |  | % within Case-Control | 28.1% | 71.9% | 100.0% |
|  |  |  | Case | Count | 61 | 99 | 160 |
|  |  |  |  | % within Case-Control | 38.1% | 61.9% | 100.0% |
|  |  | Total | | Count | 106 | 214 | 320 |
|  |  |  |  | % within Case-Control | 33.1% | 66.9% | 100.0% |
| 2 | < 6 hours | Case-Control | Control | Count | 12 | 54 | 66 |
|  |  |  |  | % within Case-Control | 18.2% | 81.8% | 100.0% |
|  |  |  | Case | Count | 26 | 42 | 68 |
|  |  |  |  | % within Case-Control | 38.2% | 61.8% | 100.0% |
|  |  | Total | | Count | 38 | 96 | 134 |
|  |  |  |  | % within Case-Control | 28.4% | 71.6% | 100.0% |
|  | >= 6 hours | Case-Control | Control | Count | 29 | 65 | 94 |
|  |  |  |  | % within Case-Control | 30.9% | 69.1% | 100.0% |
|  |  |  | Case | Count | 40 | 52 | 92 |
|  |  |  |  | % within Case-Control | 43.5% | 56.5% | 100.0% |
|  |  | Total | | Count | 69 | 117 | 186 |
|  |  |  |  | % within Case-Control | 37.1% | 62.9% | 100.0% |
|  | Total | Case-Control | Control | Count | 41 | 119 | 160 |
|  |  |  |  | % within Case-Control | 25.6% | 74.4% | 100.0% |
|  |  |  | Case | Count | 66 | 94 | 160 |
|  |  |  |  | % within Case-Control | 41.3% | 58.8% | 100.0% |
|  |  | Total | | Count | 107 | 213 | 320 |
|  |  |  |  | % within Case-Control | 33.4% | 66.6% | 100.0% |
| 3 | < 6 hours | Case-Control | Control | Count | 14 | 52 | 66 |
|  |  |  |  | % within Case-Control | 21.2% | 78.8% | 100.0% |
|  |  |  | Case | Count | 24 | 47 | 71 |
|  |  |  |  | % within Case-Control | 33.8% | 66.2% | 100.0% |
|  |  | Total | | Count | 38 | 99 | 137 |
|  |  |  |  | % within Case-Control | 27.7% | 72.3% | 100.0% |
|  | >= 6 hours | Case-Control | Control | Count | 30 | 64 | 94 |
|  |  |  |  | % within Case-Control | 31.9% | 68.1% | 100.0% |
|  |  |  | Case | Count | 39 | 50 | 89 |
|  |  |  |  | % within Case-Control | 43.8% | 56.2% | 100.0% |
|  |  | Total | | Count | 69 | 114 | 183 |
|  |  |  |  | % within Case-Control | 37.7% | 62.3% | 100.0% |
|  | Total | Case-Control | Control | Count | 44 | 116 | 160 |
|  |  |  |  | % within Case-Control | 27.5% | 72.5% | 100.0% |
|  |  |  | Case | Count | 63 | 97 | 160 |
|  |  |  |  | % within Case-Control | 39.4% | 60.6% | 100.0% |
|  |  | Total | | Count | 107 | 213 | 320 |
|  |  |  |  | % within Case-Control | 33.4% | 66.6% | 100.0% |
| 4 | < 6 hours | Case-Control | Control | Count | 15 | 51 | 66 |
|  |  |  |  | % within Case-Control | 22.7% | 77.3% | 100.0% |
|  |  |  | Case | Count | 21 | 49 | 70 |
|  |  |  |  | % within Case-Control | 30.0% | 70.0% | 100.0% |
|  |  | Total | | Count | 36 | 100 | 136 |
|  |  |  |  | % within Case-Control | 26.5% | 73.5% | 100.0% |
|  | >= 6 hours | Case-Control | Control | Count | 29 | 65 | 94 |
|  |  |  |  | % within Case-Control | 30.9% | 69.1% | 100.0% |
|  |  |  | Case | Count | 42 | 48 | 90 |
|  |  |  |  | % within Case-Control | 46.7% | 53.3% | 100.0% |
|  |  | Total | | Count | 71 | 113 | 184 |
|  |  |  |  | % within Case-Control | 38.6% | 61.4% | 100.0% |
|  | Total | Case-Control | Control | Count | 44 | 116 | 160 |
|  |  |  |  | % within Case-Control | 27.5% | 72.5% | 100.0% |
|  |  |  | Case | Count | 63 | 97 | 160 |
|  |  |  |  | % within Case-Control | 39.4% | 60.6% | 100.0% |
|  |  | Total | | Count | 107 | 213 | 320 |
|  |  |  |  | % within Case-Control | 33.4% | 66.6% | 100.0% |
| 5 | < 6 hours | Case-Control | Control | Count | 13 | 53 | 66 |
|  |  |  |  | % within Case-Control | 19.7% | 80.3% | 100.0% |
|  |  |  | Case | Count | 22 | 50 | 72 |
|  |  |  |  | % within Case-Control | 30.6% | 69.4% | 100.0% |
|  |  | Total | | Count | 35 | 103 | 138 |
|  |  |  |  | % within Case-Control | 25.4% | 74.6% | 100.0% |
|  | >= 6 hours | Case-Control | Control | Count | 32 | 62 | 94 |
|  |  |  |  | % within Case-Control | 34.0% | 66.0% | 100.0% |
|  |  |  | Case | Count | 40 | 48 | 88 |
|  |  |  |  | % within Case-Control | 45.5% | 54.5% | 100.0% |
|  |  | Total | | Count | 72 | 110 | 182 |
|  |  |  |  | % within Case-Control | 39.6% | 60.4% | 100.0% |
|  | Total | Case-Control | Control | Count | 45 | 115 | 160 |
|  |  |  |  | % within Case-Control | 28.1% | 71.9% | 100.0% |
|  |  |  | Case | Count | 62 | 98 | 160 |
|  |  |  |  | % within Case-Control | 38.8% | 61.3% | 100.0% |
|  |  | Total | | Count | 107 | 213 | 320 |
|  |  |  |  | % within Case-Control | 33.4% | 66.6% | 100.0% |
| Pooled | < 6 hours | Case-Control | Control | Count | 13 | 53 | 66 |
|  |  |  | Case | Count | 22 | 48 | 70 |
|  |  | Total | | Count | 35 | 101 | 136 |
|  | >= 6 hours | Case-Control | Control | Count | 30.8 | 63.2 | 94 |
|  |  |  | Case | Count | 41 | 49 | 90 |
|  |  | Total | | Count | 71.8 | 112.2 | 184 |
|  | Total | Case-Control | Control | Count | 43.8 | 116.2 | 160 |
|  |  |  | Case | Count | 63 | 97 | 160 |
|  |  | Total | | Count | 106.8 | 213.2 | 320 |

**Chi-Square Test Case-Control and Income break down by Hours Worked (using pooled frequency)**

| **Case-Control * Income (dichotom) * Hours Worked Cross-tabulation** | | | | | | |
| --- | --- | --- | --- | --- | --- | --- |
| Hours Worked | | | | Income (dichotom) | | Total |
|  |  |  |  | High Income | Lower Middle Income |  |
| < 6 hours | Case-Control | Control | Count | 13.0 | 53.0 | 66.0 |
|  |  |  | % within Case-Control | 19.7% | 80.3% | 100.0% |
|  |  | Case | Count | 22.0 | 48.0 | 70.0 |
|  |  |  | % within Case-Control | 31.4% | 68.6% | 100.0% |
|  | Total | | Count | 35.0 | 101.0 | 136.0 |
|  |  |  | % within Case-Control | 25.7% | 74.3% | 100.0% |
| >= 6 hours | Case-Control | Control | Count | 30.8 | 63.2 | 94.0 |
|  |  |  | % within Case-Control | 32.8% | 67.2% | 100.0% |
|  |  | Case | Count | 41.0 | 49.0 | 90.0 |
|  |  |  | % within Case-Control | 45.6% | 54.4% | 100.0% |
|  | Total | | Count | 71.8 | 112.2 | 184.0 |
|  |  |  | % within Case-Control | 39.0% | 61.0% | 100.0% |
| Total | Case-Control | Control | Count | 43.8 | 116.2 | 160.0 |
|  |  |  | % within Case-Control | 27.4% | 72.6% | 100.0% |
|  |  | Case | Count | 63.0 | 97.0 | 160.0 |
|  |  |  | % within Case-Control | 39.4% | 60.6% | 100.0% |
|  | Total | | Count | 106.8 | 213.2 | 320.0 |
|  |  |  | % within Case-Control | 33.4% | 66.6% | 100.0% |

| **Chi-Square Tests** | | | | | | |
| --- | --- | --- | --- | --- | --- | --- |
| Hours Worked | | Value | df | Asymptotic Significance (2-sided) | Exact Sig. (2-sided) | Exact Sig. (1-sided) |
| < 6 hours | Pearson Chi-Square | 2.446^c^ | 1 | .118 |  |  |
|  | Continuity Correction^b^ | 1.871 | 1 | .171 |  |  |
|  | Likelihood Ratio | 2.470 | 1 | .116 |  |  |
|  | Fisher's Exact Test |  |  |  | .169 | .085 |
|  | Linear-by-Linear Association | 2.428 | 1 | .119 |  |  |
|  | N of Valid Cases | 136 |  |  |  |  |
| >= 6 hours | Pearson Chi-Square | 3.161^d^ | 1 | .075 |  |  |
|  | Continuity Correction^b^ | 2.646 | 1 | .104 |  |  |
|  | Likelihood Ratio | 3.169 | 1 | .075 |  |  |
|  | Fisher's Exact Test |  |  |  | .097 | .055 |
|  | Linear-by-Linear Association | 3.144 | 1 | .076 |  |  |
|  | N of Valid Cases | 184 |  |  |  |  |
| Total | Pearson Chi-Square | 5.181^a^ | 1 | .023 |  |  |
|  | Continuity Correction^b^ | 4.655 | 1 | .031 |  |  |
|  | Likelihood Ratio | 5.202 | 1 | .023 |  |  |
|  | Fisher's Exact Test |  |  |  | .033 | .016 |
|  | Linear-by-Linear Association | 5.165 | 1 | .023 |  |  |
|  | N of Valid Cases | 320 |  |  |  |  |
| a. 0 cells (0.0%) have expected count less than 5. The minimum expected count is 53.40. | | | | | | |
| b. Computed only for a 2x2 table | | | | | | |
| c. 0 cells (0.0%) have expected count less than 5. The minimum expected count is 16.99. | | | | | | |
| d. 0 cells (0.0%) have expected count less than 5. The minimum expected count is 35.12. | | | | | | |

1. **Cross-tabulation of Case-Control and Income break down by Cohabitating family members**

| **Case-Control * Income (dichotom) * Cohabitating family members Cross-tabulation** | | | | | | | |
| --- | --- | --- | --- | --- | --- | --- | --- |
| Imputation Number | Cohabitating family members | | | | Income (dichotom) | | Total |
|  |  |  |  |  | High Income | Lower Middle Income |  |
| Original data | with 0 | Case-Control | Control | Count | 3 | 8 | 11 |
|  |  |  |  | % within Case-Control | 27.3% | 72.7% | 100.0% |
|  |  |  | Case | Count | 0 | 2 | 2 |
|  |  |  |  | % within Case-Control | 0.0% | 100.0% | 100.0% |
|  |  | Total | | Count | 3 | 10 | 13 |
|  |  |  |  | % within Case-Control | 23.1% | 76.9% | 100.0% |
|  | with 1-3 | Case-Control | Control | Count | 20 | 48 | 68 |
|  |  |  |  | % within Case-Control | 29.4% | 70.6% | 100.0% |
|  |  |  | Case | Count | 17 | 26 | 43 |
|  |  |  |  | % within Case-Control | 39.5% | 60.5% | 100.0% |
|  |  | Total | | Count | 37 | 74 | 111 |
|  |  |  |  | % within Case-Control | 33.3% | 66.7% | 100.0% |
|  | with >=4 | Case-Control | Control | Count | 10 | 43 | 53 |
|  |  |  |  | % within Case-Control | 18.9% | 81.1% | 100.0% |
|  |  |  | Case | Count | 17 | 27 | 44 |
|  |  |  |  | % within Case-Control | 38.6% | 61.4% | 100.0% |
|  |  | Total | | Count | 27 | 70 | 97 |
|  |  |  |  | % within Case-Control | 27.8% | 72.2% | 100.0% |
|  | Total | Case-Control | Control | Count | 33 | 99 | 132 |
|  |  |  |  | % within Case-Control | 25.0% | 75.0% | 100.0% |
|  |  |  | Case | Count | 34 | 55 | 89 |
|  |  |  |  | % within Case-Control | 38.2% | 61.8% | 100.0% |
|  |  | Total | | Count | 67 | 154 | 221 |
|  |  |  |  | % within Case-Control | 30.3% | 69.7% | 100.0% |
| 1 | with 0 | Case-Control | Control | Count | 3 | 9 | 12 |
|  |  |  |  | % within Case-Control | 25.0% | 75.0% | 100.0% |
|  |  |  | Case | Count | 3 | 4 | 7 |
|  |  |  |  | % within Case-Control | 42.9% | 57.1% | 100.0% |
|  |  | Total | | Count | 6 | 13 | 19 |
|  |  |  |  | % within Case-Control | 31.6% | 68.4% | 100.0% |
|  | with 1-3 | Case-Control | Control | Count | 26 | 60 | 86 |
|  |  |  |  | % within Case-Control | 30.2% | 69.8% | 100.0% |
|  |  |  | Case | Count | 27 | 45 | 72 |
|  |  |  |  | % within Case-Control | 37.5% | 62.5% | 100.0% |
|  |  | Total | | Count | 53 | 105 | 158 |
|  |  |  |  | % within Case-Control | 33.5% | 66.5% | 100.0% |
|  | with >=4 | Case-Control | Control | Count | 16 | 46 | 62 |
|  |  |  |  | % within Case-Control | 25.8% | 74.2% | 100.0% |
|  |  |  | Case | Count | 31 | 50 | 81 |
|  |  |  |  | % within Case-Control | 38.3% | 61.7% | 100.0% |
|  |  | Total | | Count | 47 | 96 | 143 |
|  |  |  |  | % within Case-Control | 32.9% | 67.1% | 100.0% |
|  | Total | Case-Control | Control | Count | 45 | 115 | 160 |
|  |  |  |  | % within Case-Control | 28.1% | 71.9% | 100.0% |
|  |  |  | Case | Count | 61 | 99 | 160 |
|  |  |  |  | % within Case-Control | 38.1% | 61.9% | 100.0% |
|  |  | Total | | Count | 106 | 214 | 320 |
|  |  |  |  | % within Case-Control | 33.1% | 66.9% | 100.0% |
| 2 | with 0 | Case-Control | Control | Count | 3 | 9 | 12 |
|  |  |  |  | % within Case-Control | 25.0% | 75.0% | 100.0% |
|  |  |  | Case | Count | 2 | 5 | 7 |
|  |  |  |  | % within Case-Control | 28.6% | 71.4% | 100.0% |
|  |  | Total | | Count | 5 | 14 | 19 |
|  |  |  |  | % within Case-Control | 26.3% | 73.7% | 100.0% |
|  | with 1-3 | Case-Control | Control | Count | 25 | 61 | 86 |
|  |  |  |  | % within Case-Control | 29.1% | 70.9% | 100.0% |
|  |  |  | Case | Count | 30 | 42 | 72 |
|  |  |  |  | % within Case-Control | 41.7% | 58.3% | 100.0% |
|  |  | Total | | Count | 55 | 103 | 158 |
|  |  |  |  | % within Case-Control | 34.8% | 65.2% | 100.0% |
|  | with >=4 | Case-Control | Control | Count | 13 | 49 | 62 |
|  |  |  |  | % within Case-Control | 21.0% | 79.0% | 100.0% |
|  |  |  | Case | Count | 34 | 47 | 81 |
|  |  |  |  | % within Case-Control | 42.0% | 58.0% | 100.0% |
|  |  | Total | | Count | 47 | 96 | 143 |
|  |  |  |  | % within Case-Control | 32.9% | 67.1% | 100.0% |
|  | Total | Case-Control | Control | Count | 41 | 119 | 160 |
|  |  |  |  | % within Case-Control | 25.6% | 74.4% | 100.0% |
|  |  |  | Case | Count | 66 | 94 | 160 |
|  |  |  |  | % within Case-Control | 41.3% | 58.8% | 100.0% |
|  |  | Total | | Count | 107 | 213 | 320 |
|  |  |  |  | % within Case-Control | 33.4% | 66.6% | 100.0% |
| 3 | with 0 | Case-Control | Control | Count | 3 | 9 | 12 |
|  |  |  |  | % within Case-Control | 25.0% | 75.0% | 100.0% |
|  |  |  | Case | Count | 2 | 5 | 7 |
|  |  |  |  | % within Case-Control | 28.6% | 71.4% | 100.0% |
|  |  | Total | | Count | 5 | 14 | 19 |
|  |  |  |  | % within Case-Control | 26.3% | 73.7% | 100.0% |
|  | with 1-3 | Case-Control | Control | Count | 27 | 59 | 86 |
|  |  |  |  | % within Case-Control | 31.4% | 68.6% | 100.0% |
|  |  |  | Case | Count | 31 | 42 | 73 |
|  |  |  |  | % within Case-Control | 42.5% | 57.5% | 100.0% |
|  |  | Total | | Count | 58 | 101 | 159 |
|  |  |  |  | % within Case-Control | 36.5% | 63.5% | 100.0% |
|  | with >=4 | Case-Control | Control | Count | 14 | 48 | 62 |
|  |  |  |  | % within Case-Control | 22.6% | 77.4% | 100.0% |
|  |  |  | Case | Count | 30 | 50 | 80 |
|  |  |  |  | % within Case-Control | 37.5% | 62.5% | 100.0% |
|  |  | Total | | Count | 44 | 98 | 142 |
|  |  |  |  | % within Case-Control | 31.0% | 69.0% | 100.0% |
|  | Total | Case-Control | Control | Count | 44 | 116 | 160 |
|  |  |  |  | % within Case-Control | 27.5% | 72.5% | 100.0% |
|  |  |  | Case | Count | 63 | 97 | 160 |
|  |  |  |  | % within Case-Control | 39.4% | 60.6% | 100.0% |
|  |  | Total | | Count | 107 | 213 | 320 |
|  |  |  |  | % within Case-Control | 33.4% | 66.6% | 100.0% |
| 4 | with 0 | Case-Control | Control | Count | 4 | 8 | 12 |
|  |  |  |  | % within Case-Control | 33.3% | 66.7% | 100.0% |
|  |  |  | Case | Count | 4 | 3 | 7 |
|  |  |  |  | % within Case-Control | 57.1% | 42.9% | 100.0% |
|  |  | Total | | Count | 8 | 11 | 19 |
|  |  |  |  | % within Case-Control | 42.1% | 57.9% | 100.0% |
|  | with 1-3 | Case-Control | Control | Count | 28 | 58 | 86 |
|  |  |  |  | % within Case-Control | 32.6% | 67.4% | 100.0% |
|  |  |  | Case | Count | 30 | 43 | 73 |
|  |  |  |  | % within Case-Control | 41.1% | 58.9% | 100.0% |
|  |  | Total | | Count | 58 | 101 | 159 |
|  |  |  |  | % within Case-Control | 36.5% | 63.5% | 100.0% |
|  | with >=4 | Case-Control | Control | Count | 12 | 50 | 62 |
|  |  |  |  | % within Case-Control | 19.4% | 80.6% | 100.0% |
|  |  |  | Case | Count | 28 | 51 | 79 |
|  |  |  |  | % within Case-Control | 35.4% | 64.6% | 100.0% |
|  |  | Total | | Count | 40 | 101 | 141 |
|  |  |  |  | % within Case-Control | 28.4% | 71.6% | 100.0% |
|  | Total | Case-Control | Control | Count | 44 | 116 | 160 |
|  |  |  |  | % within Case-Control | 27.5% | 72.5% | 100.0% |
|  |  |  | Case | Count | 62 | 97 | 159 |
|  |  |  |  | % within Case-Control | 39.0% | 61.0% | 100.0% |
|  |  | Total | | Count | 106 | 213 | 319 |
|  |  |  |  | % within Case-Control | 33.2% | 66.8% | 100.0% |
| 5 | with 0 | Case-Control | Control | Count | 3 | 9 | 12 |
|  |  |  |  | % within Case-Control | 25.0% | 75.0% | 100.0% |
|  |  |  | Case | Count | 1 | 6 | 7 |
|  |  |  |  | % within Case-Control | 14.3% | 85.7% | 100.0% |
|  |  | Total | | Count | 4 | 15 | 19 |
|  |  |  |  | % within Case-Control | 21.1% | 78.9% | 100.0% |
|  | with 1-3 | Case-Control | Control | Count | 29 | 57 | 86 |
|  |  |  |  | % within Case-Control | 33.7% | 66.3% | 100.0% |
|  |  |  | Case | Count | 30 | 44 | 74 |
|  |  |  |  | % within Case-Control | 40.5% | 59.5% | 100.0% |
|  |  | Total | | Count | 59 | 101 | 160 |
|  |  |  |  | % within Case-Control | 36.9% | 63.1% | 100.0% |
|  | with >=4 | Case-Control | Control | Count | 13 | 49 | 62 |
|  |  |  |  | % within Case-Control | 21.0% | 79.0% | 100.0% |
|  |  |  | Case | Count | 31 | 48 | 79 |
|  |  |  |  | % within Case-Control | 39.2% | 60.8% | 100.0% |
|  |  | Total | | Count | 44 | 97 | 141 |
|  |  |  |  | % within Case-Control | 31.2% | 68.8% | 100.0% |
|  | Total | Case-Control | Control | Count | 45 | 115 | 160 |
|  |  |  |  | % within Case-Control | 28.1% | 71.9% | 100.0% |
|  |  |  | Case | Count | 62 | 98 | 160 |
|  |  |  |  | % within Case-Control | 38.8% | 61.3% | 100.0% |
|  |  | Total | | Count | 107 | 213 | 320 |
|  |  |  |  | % within Case-Control | 33.4% | 66.6% | 100.0% |
| Pooled | with 0 | Case-Control | Control | Count | 3.2 | 8.8 | 12 |
|  |  |  | Case | Count | 2.4 | 4.6 | 7 |
|  |  | Total | | Count | 5.6 | 13.4 | 19 |
|  | with 1-3 | Case-Control | Control | Count | 27 | 59 | 86 |
|  |  |  | Case | Count | 29.6 | 43.2 | 72.8 |
|  |  | Total | | Count | 56.6 | 102.2 | 158.8 |
|  | with >=4 | Case-Control | Control | Count | 13.6 | 48.4 | 62 |
|  |  |  | Case | Count | 30.8 | 49.2 | 80 |
|  |  | Total | | Count | 44.4 | 97.6 | 142 |
|  | Total | Case-Control | Control | Count | 43.8 | 116.2 | 160 |
|  |  |  | Case | Count | 62.8 | 97 | 159.8 |
|  |  | Total | | Count | 106.6 | 213.2 | 319.8 |

**Chi-Square Test Case-Control and Income break down by Cohabitating family members (using pooled frequency)**

| **Case-Control * Income (dichotom) * Cohabitating family members Crosstabulation** | | | | | | |
| --- | --- | --- | --- | --- | --- | --- |
| Cohabitating family members | | | | Income (dichotom) | | Total |
|  |  |  |  | High Income | Lower Middle Income |  |
| with 0 | Case-Control | Control | Count | 3.2 | 8.8 | 12.0 |
|  |  |  | % within Case-Control | 26.7% | 73.3% | 100.0% |
|  |  | Case | Count | 2.4 | 4.6 | 7.0 |
|  |  |  | % within Case-Control | 34.3% | 65.7% | 100.0% |
|  | Total | | Count | 5.6 | 13.4 | 19.0 |
|  |  |  | % within Case-Control | 29.5% | 70.5% | 100.0% |
| with 1-3 | Case-Control | Control | Count | 27.0 | 59.0 | 86.0 |
|  |  |  | % within Case-Control | 31.4% | 68.6% | 100.0% |
|  |  | Case | Count | 29.6 | 43.2 | 72.8 |
|  |  |  | % within Case-Control | 40.7% | 59.3% | 100.0% |
|  | Total | | Count | 56.6 | 102.2 | 158.8 |
|  |  |  | % within Case-Control | 35.6% | 64.4% | 100.0% |
| with >=4 | Case-Control | Control | Count | 13.6 | 48.4 | 62.0 |
|  |  |  | % within Case-Control | 21.9% | 78.1% | 100.0% |
|  |  | Case | Count | 30.8 | 49.2 | 80.0 |
|  |  |  | % within Case-Control | 38.5% | 61.5% | 100.0% |
|  | Total | | Count | 44.4 | 97.6 | 142.0 |
|  |  |  | % within Case-Control | 31.3% | 68.7% | 100.0% |
| Total | Case-Control | Control | Count | 43.8 | 116.2 | 160.0 |
|  |  |  | % within Case-Control | 27.4% | 72.6% | 100.0% |
|  |  | Case | Count | 62.8 | 97.0 | 159.8 |
|  |  |  | % within Case-Control | 39.3% | 60.7% | 100.0% |
|  | Total | | Count | 106.6 | 213.2 | 319.8 |
|  |  |  | % within Case-Control | 33.3% | 66.7% | 100.0% |

| **Chi-Square Tests** | | | | | | |
| --- | --- | --- | --- | --- | --- | --- |
| Cohabitating family members | | Value | df | Asymptotic Significance (2-sided) | Exact Sig. (2-sided) | Exact Sig. (1-sided) |
| with 0 | Pearson Chi-Square | .123^c^ | 1 | .725 |  |  |
|  | Continuity Correction^b^ | .000 | 1 | 1.000 |  |  |
|  | Likelihood Ratio | .122 | 1 | .727 |  |  |
|  | Fisher's Exact Test |  |  |  | 1.000 | .634 |
|  | Linear-by-Linear Association | .117 | 1 | .732 |  |  |
|  | N of Valid Cases | 19 |  |  |  |  |
| with 1-3 | Pearson Chi-Square | 1.475^d^ | 1 | .225 |  |  |
|  | Continuity Correction^b^ | 1.099 | 1 | .295 |  |  |
|  | Likelihood Ratio | 1.473 | 1 | .225 |  |  |
|  | Fisher's Exact Test |  |  |  | .246 | .135 |
|  | Linear-by-Linear Association | 1.466 | 1 | .226 |  |  |
|  | N of Valid Cases | 159 |  |  |  |  |
| with >=4 | Pearson Chi-Square | 4.460^e^ | 1 | .035 |  |  |
|  | Continuity Correction^b^ | 3.722 | 1 | .054 |  |  |
|  | Likelihood Ratio | 4.559 | 1 | .033 |  |  |
|  | Fisher's Exact Test |  |  |  | .047 | .030 |
|  | Linear-by-Linear Association | 4.428 | 1 | .035 |  |  |
|  | N of Valid Cases | 142 |  |  |  |  |
| Total | Pearson Chi-Square | 5.115^a^ | 1 | .024 |  |  |
|  | Continuity Correction^b^ | 4.593 | 1 | .032 |  |  |
|  | Likelihood Ratio | 5.136 | 1 | .023 |  |  |
|  | Fisher's Exact Test |  |  |  | .033 | .016 |
|  | Linear-by-Linear Association | 5.099 | 1 | .024 |  |  |
|  | N of Valid Cases | 320 |  |  |  |  |
| a. 0 cells (0.0%) have expected count less than 5. The minimum expected count is 53.27. | | | | | | |
| b. Computed only for a 2x2 table | | | | | | |
| c. 3 cells (75.0%) have expected count less than 5. The minimum expected count is 2.06. | | | | | | |
| d. 0 cells (0.0%) have expected count less than 5. The minimum expected count is 25.95. | | | | | | |
| e. 0 cells (0.0%) have expected count less than 5. The minimum expected count is 19.39. | | | | | | |
