# Supplementary material for: Socio-economic-demographic determinants of depression in Indonesia: A hospital-based study
Source: PLoS One. 2020 Dec 15;15(12):e0244108. doi: 10.1371/journal.pone.0244108 (PMC7737985; doi:10.1371/journal.pone.0244108)
Supplement: S4 Appendix — (DOCX) [file pone.0244108.s004.docx]

**S4 Appendix. Original and imputation results for socio-economic-demographic variables with missing value in Table 6**

**Multiple Logistic Regression Analysis**

| **Variables in the Equation** | | | | | | | | | | | | | |
| --- | --- | --- | --- | --- | --- | --- | --- | --- | --- | --- | --- | --- | --- |
| Imputation Number | | | B | S.E. | Wald | df | Sig. | Exp(B) | 95% C.I.for EXP(B) | | Fraction Missing Info. | Relative Increase Variance | Relative Efficiency |
|  |  |  |  |  |  |  |  |  | Lower | Upper |  |  |  |
| Original data | Step 1^a^ | Age (dichotom) | .690 | .371 | 3.463 | 1 | .063 | 1.993 | .964 | 4.123 |  |  |  |
|  |  | Sex^b^ | -.115 | .326 | .125 | 1 | .724 | .891 | .470 | 1.690 |  |  |  |
|  |  | Ethnicity (dichotom)^b^ | -.549 | .376 | 2.127 | 1 | .145 | .578 | .276 | 1.208 |  |  |  |
|  |  | Education (dichotom) | .346 | .344 | 1.013 | 1 | .314 | 1.413 | .721 | 2.771 |  |  |  |
|  |  | Income (dichotom) | .774 | .361 | 4.600 | 1 | .032 | 2.168 | 1.069 | 4.398 |  |  |  |
|  |  | Occupation (dichotom) | .232 | .355 | .426 | 1 | .514 | 1.261 | .629 | 2.527 |  |  |  |
|  |  | Hours Worked | -.466 | .360 | 1.674 | 1 | .196 | .627 | .310 | 1.271 |  |  |  |
|  |  | Cohabitating Family Members (dichotom) | .452 | .316 | 2.050 | 1 | .152 | 1.571 | .846 | 2.916 |  |  |  |
|  |  | Constant | -1.019 | .456 | 5.001 | 1 | .025 | .361 |  |  |  |  |  |
| 1 | Step 1^a^ | Age (dichotom) | .350 | .282 | 1.537 | 1 | .215 | 1.419 | .816 | 2.469 |  |  |  |
|  |  | Sex^b^ | -.244 | .255 | .920 | 1 | .337 | .783 | .475 | 1.290 |  |  |  |
|  |  | Ethnicity (dichotom)^b^ | -.359 | .270 | 1.769 | 1 | .183 | .699 | .412 | 1.185 |  |  |  |
|  |  | Education (dichotom) | .502 | .243 | 4.245 | 1 | .039 | 1.651 | 1.025 | 2.661 |  |  |  |
|  |  | Income (dichotom) | .638 | .263 | 5.873 | 1 | .015 | 1.894 | 1.130 | 3.173 |  |  |  |
|  |  | Occupation (dichotom) | -.315 | .261 | 1.457 | 1 | .227 | .730 | .438 | 1.217 |  |  |  |
|  |  | Hours Worked | -.097 | .260 | .139 | 1 | .709 | .908 | .545 | 1.511 |  |  |  |
|  |  | Cohabitating Family Members (dichotom) | .478 | .234 | 4.171 | 1 | .041 | 1.613 | 1.019 | 2.554 |  |  |  |
|  |  | Constant | -.266 | .352 | .572 | 1 | .450 | .766 |  |  |  |  |  |
| 2 | Step 1^a^ | Age (dichotom) | .391 | .287 | 1.862 | 1 | .172 | 1.479 | .843 | 2.595 |  |  |  |
|  |  | Sex^b^ | -.265 | .256 | 1.073 | 1 | .300 | .767 | .465 | 1.267 |  |  |  |
|  |  | Ethnicity (dichotom)^b^ | -.329 | .273 | 1.454 | 1 | .228 | .720 | .421 | 1.229 |  |  |  |
|  |  | Education (dichotom) | .564 | .247 | 5.225 | 1 | .022 | 1.757 | 1.084 | 2.850 |  |  |  |
|  |  | Income (dichotom) | .912 | .263 | 11.994 | 1 | .001 | 2.489 | 1.486 | 4.171 |  |  |  |
|  |  | Occupation (dichotom) | -.372 | .264 | 1.977 | 1 | .160 | .690 | .411 | 1.158 |  |  |  |
|  |  | Hours Worked | .006 | .260 | .000 | 1 | .983 | 1.006 | .605 | 1.673 |  |  |  |
|  |  | Cohabitating Family Members (dichotom) | .495 | .238 | 4.337 | 1 | .037 | 1.641 | 1.030 | 2.615 |  |  |  |
|  |  | Constant | -.428 | .357 | 1.437 | 1 | .231 | .652 |  |  |  |  |  |
| 3 | Step 1^a^ | Age (dichotom) | .451 | .283 | 2.539 | 1 | .111 | 1.570 | .901 | 2.734 |  |  |  |
|  |  | Sex^b^ | -.236 | .253 | .875 | 1 | .350 | .789 | .481 | 1.296 |  |  |  |
|  |  | Ethnicity (dichotom)^b^ | -.356 | .271 | 1.724 | 1 | .189 | .701 | .412 | 1.191 |  |  |  |
|  |  | Education (dichotom) | .507 | .243 | 4.345 | 1 | .037 | 1.661 | 1.031 | 2.675 |  |  |  |
|  |  | Income (dichotom) | .726 | .258 | 7.926 | 1 | .005 | 2.067 | 1.247 | 3.425 |  |  |  |
|  |  | Occupation (dichotom) | -.247 | .259 | .913 | 1 | .339 | .781 | .470 | 1.297 |  |  |  |
|  |  | Hours Worked | -.086 | .256 | .111 | 1 | .739 | .918 | .555 | 1.517 |  |  |  |
|  |  | Cohabitating Family Members (dichotom) | .501 | .236 | 4.501 | 1 | .034 | 1.650 | 1.039 | 2.621 |  |  |  |
|  |  | Constant | -.379 | .355 | 1.142 | 1 | .285 | .684 |  |  |  |  |  |
| 4 | Step 1^a^ | Age (dichotom) | .390 | .285 | 1.874 | 1 | .171 | 1.477 | .845 | 2.582 |  |  |  |
|  |  | Sex^b^ | -.215 | .254 | .720 | 1 | .396 | .806 | .490 | 1.326 |  |  |  |
|  |  | Ethnicity (dichotom)^b^ | -.316 | .271 | 1.359 | 1 | .244 | .729 | .428 | 1.240 |  |  |  |
|  |  | Education (dichotom) | .540 | .245 | 4.875 | 1 | .027 | 1.716 | 1.063 | 2.771 |  |  |  |
|  |  | Income (dichotom) | .762 | .263 | 8.380 | 1 | .004 | 2.143 | 1.279 | 3.591 |  |  |  |
|  |  | Occupation (dichotom) | -.258 | .262 | .975 | 1 | .323 | .772 | .462 | 1.290 |  |  |  |
|  |  | Hours Worked | -.095 | .257 | .136 | 1 | .712 | .910 | .550 | 1.506 |  |  |  |
|  |  | Cohabitating Family Members (dichotom) | .514 | .237 | 4.686 | 1 | .030 | 1.672 | 1.050 | 2.663 |  |  |  |
|  |  | Constant | -.411 | .359 | 1.310 | 1 | .252 | .663 |  |  |  |  |  |
| 5 | Step 1^a^ | Age (dichotom) | .324 | .282 | 1.320 | 1 | .251 | 1.382 | .796 | 2.401 |  |  |  |
|  |  | Sex^b^ | -.255 | .254 | 1.009 | 1 | .315 | .775 | .471 | 1.275 |  |  |  |
|  |  | Ethnicity (dichotom) ^b^ | -.294 | .271 | 1.178 | 1 | .278 | .745 | .438 | 1.267 |  |  |  |
|  |  | Education (dichotom) | .506 | .243 | 4.344 | 1 | .037 | 1.659 | 1.031 | 2.669 |  |  |  |
|  |  | Income (dichotom) | .641 | .261 | 6.040 | 1 | .014 | 1.899 | 1.139 | 3.167 |  |  |  |
|  |  | Occupation (dichotom) | -.254 | .262 | .940 | 1 | .332 | .776 | .465 | 1.296 |  |  |  |
|  |  | Hours Worked | -.158 | .258 | .373 | 1 | .541 | .854 | .515 | 1.417 |  |  |  |
|  |  | Cohabitating Family Members (dichotom) | .460 | .235 | 3.847 | 1 | .050 | 1.584 | 1.000 | 2.509 |  |  |  |
|  |  | Constant | -.259 | .356 | .529 | 1 | .467 | .772 |  |  |  |  |  |
| Pooled | Step 1^a^ | Age (dichotom) | .381 | .289 |  |  | .187 | 1.464 | .831 | 2.579 | .034 | .035 | .993 |
|  |  | Sex^b^ | -.243 | .255 |  |  | .340 | .784 | .476 | 1.293 | .007 | .007 | .999 |
|  |  | Ethnicity (dichotom)^b^ | -.331 | .273 |  |  | .225 | .718 | .421 | 1.226 | .012 | .012 | .998 |
|  |  | Education (dichotom) | .524 | .246 |  |  | .033 | 1.688 | 1.042 | 2.734 | .015 | .015 | .997 |
|  |  | Income (dichotom) | .736 | .289 |  |  | .012 | 2.088 | 1.178 | 3.700 | .193 | .220 | .963 |
|  |  | Occupation (dichotom) | -.289 | .268 |  |  | .281 | .749 | .443 | 1.267 | .049 | .050 | .990 |
|  |  | Hours Worked | -.086 | .266 |  |  | .747 | .918 | .545 | 1.547 | .060 | .062 | .988 |
|  |  | Cohabitating Family Members (dichotom) | .490 | .237 |  |  | .039 | 1.632 | 1.025 | 2.597 | .009 | .009 | .998 |
|  |  | Constant | -.349 | .367 |  |  | .342 | .706 | .344 | 1.449 | .060 | .062 | .988 |
| 1. Variable(s) entered on step 1: Age (dichotom), Sex, Ethnicity (dichotom), Education (dichotom), Income (dichotom), Occupation (dichotom), Hours Worked , Cohabitating Family Members (dichotom). 2. No missing in both variables on this analysis | | | | | | | | | | | | | |
