# Supplementary material for: Socio-economic-demographic determinants of depression in Indonesia: A hospital-based study
Source: PLoS One. 2020 Dec 15;15(12):e0244108. doi: 10.1371/journal.pone.0244108 (PMC7737985; doi:10.1371/journal.pone.0244108)
Supplement: S5 Appendix — (DOCX) [file pone.0244108.s005.docx]

**S5 Appendix. Form for basic information interview (Indonesian and English version)**

*Lembar Informasi dasar*

**Informasi Partisipan**

| ID |  | | | | | Nama pewawancara | | | |  | | | | | | |
| --- | --- | --- | --- | --- | --- | --- | --- | --- | --- | --- | --- | --- | --- | --- | --- | --- |
|  |  |  |  |  |  | Tanggal wawancara (DD/MM/YYYY) | | | | | | | / / | | | |
| Nama | | | (Nama asli) | | | | | (Nama keluarga) | | | | | | (Lainnya) | | |
| Tanggal lahir | | | | | (Tahun) | | | | (Bulan) | | (Hari) | | | | Usia |  |
| Tempat tinggal | | | | | |  | | | | | | | | | | |
| Apakah anda berpindah ketempat tinggal anda sekarang? | | | | | | Kapan? | | | | | | | | | | |
|  |  |  |  |  |  | Dimana anda tinggal sebelum pindah ke tempat tinggal yang sekarang? | | | | | | | | | | |
| Tempat Lahir | | | | | |  | | | | | | | | | | |
| Jenis Kelamin | | ❑ Perempuan🡪  ❑ Laki-laki | | | | | (Jika perempuan)  Apakah anda sedang hamil? | | | | | ❑ Ya ( bulan)  ❑ Tidak | | | | |
|  |  |  |  |  |  |  | Apakah anda sedang menyusui? | | | | | ❑ Ya  ❑ Tidak | | | | |
| Pendidikan Terakhir | | | | ❑ Pascasarjana (S2, S3)  ❑ Sarjana (S1)  ❑ Diploma (S0 (D1 – D4))  ❑ Sekolah Menengah Atas (SMA)  ❑ Sekolah Menengah Pertama (SMP)  ❑ Sekolah Dasar (SD) atau kurang | | | | | | | | | | | | |

**Pertanyaan 1. Kami ingin mengetahui tentang latar belakang etnis Anda.**

Q1-1. Berasal dari kelompok etnis manakah anda saat ini?

|  |
| --- |

Q1-2. Apakah etnis bapak anda?

|  |
| --- |

Q1-3. Apakah etnis ibu anda?

|  |
| --- |

Q1-4. Apakah etnis anda dalam tiga kurun generasi?

|  |
| --- |

Note: Orang Indonesia biasanya mengenali etnis mereka berdasarkan silsilah (misalnya, perkawinan antar kelompok etnis yang berbeda) sejak kakek buyut mereka (atau generasi pertama).

Q1-5. Bahasa apa yang anda gunakan (tolong sebutkan semuanya)?

|  |
| --- |

Q1-6. Bahasa apa yang bapak anda gunakan (tolong sebutkan semuanya)?

|  |
| --- |

Q1-6. Bahasa apa yang ibu anda gunakan (tolong sebutkan semuanya)?

|  |
| --- |

**Pertanyaan 2. Kami ingin mengetahui tentang latar belakang agama Anda.**

Q2-1. Apakah agama yang anda anut? Note: Jawaban anda akan kami rahasiakan!

❑ Islam ❑ Protestan ❑ Katolik ❑ Hindu ❑ Buddha ❑ Konfusius

❑ Lainnya ( )

Q2-2. Apakah anda menganut kepercayaan lokal yang saat ini masih eksis?

❑ Ya (Sebutkan apa nama kepercayaan tersebut? )

❑ Tidak

**Pertanyaan 3. Kami ingin mengetahui tentang latar belakang pekerjaan Anda.**

Q3-1. Apa pekerjaan Anda?

|  |
| --- |

Q3-2. Apakah Anda/anggota keluarga Anda bertani?

❑ Ya

Jika ya, apakah Anda/anggota keluarga Anda menjual hasil pertanian?

❑ Ya ❑ Tidak

❑ Tidak

Q3-3. Apakah Anda/anggota keluarga Anda menangkap ikan (termasuk dengan metode apa pun)?

❑ Ya

Jika ya, apakah Anda/anggota keluarga Anda menjual hasil tangkapan tersebut?

❑ Ya ❑ Tidak

❑ Tidak

Q3-4. Apakah Anda/anggota keluarga Anda bekerja pada perusahaan/pemerintahan/lainnya?

❑ Ya ❑ Tidak

Q3-5. Apakah Anda/anggota keluarga Anda mengelola bisnis (contoh: pengelola toko)?

❑ Ya ❑ Tidak

Q3-6. Berapa jam rata-rata Anda bekerja per hari?

❑ < 1 jam ❑ 1–2 jam ❑ 2-4 jam ❑ 4-6 jam ❑ 6-8 jam ❑ 8-10 jam ❑ > 10 jam

**Pertanyaan 4. Kami ingin mengetahui tentang latar belakang pendapatan Anda.**

**Q4-1**. Berapa pendapatan Anda dalam satu tahun terakhir?

|  |
| --- |

**Q4-2**. Berapa rata-rata pendapatan rumah tangga Anda dalam satu bulan?

❑ < Rp1.000.000 ❑ Rp1.000.000 – 2.000.000 ❑ Rp2.000.000 – 3.000.000

❑ Rp3.000.000 – 5.000.000 ❑ Rp5.000.000 – 7.500.000 ❑ Rp7.500.000 – 10.000.000

❑ > Rp10.000.000 ❑ Tidak tahu

**Pertanyaan 5. Kami ingin mengetahui tentang lingkungan keluarga Anda.**

**Q5-1**. Dengan siapa Anda tinggal sekarang?

❑ Sendiri

(*Jika Anda tidak tinggal sendiri, pilihlah semua*)

❑ Suami/istri

❑ Ayah

❑ Ibu

❑ Ayah mertua

❑ Ibu mertua

❑ Anak laki-laki/menantu laki-laki (berapa banyak? )

❑ Anak perempuan/menantu perempuan (berapa banyak? )

❑ Saudara kandung/ipar laki-laki (berapa banyak? )

❑ Saudara kandung/ipar perempuan (berapa banyak? )

❑ Kakek dan nenek/kakek dan nenek mertua (berapa banyak? )

❑ Cucu/cucu menantu (berapa banyak? )

❑ Kerabat lainnya (berapa banyak? )

❑ Tunangan/pacar

❑ Teman (berapa banyak? )

❑ Kolega/Rekan Kerja (berapa banyak? )

❑ Penjaga rumah/pembantu rumah (berapa banyak? )

❑ Lainnya (berapa banyak? )

**Rekam Medis (diisi oleh dokter psikiatri)**

Gejala depresi:

Diagnosa:

Mulai gejala:

Resep:

*Basic Information Sheet (English translation)*

**Participant Information**

| ID |  | | | | | | Interviewer’s name | | | |  | | | | | | |
| --- | --- | --- | --- | --- | --- | --- | --- | --- | --- | --- | --- | --- | --- | --- | --- | --- | --- |
|  |  |  |  |  |  |  | Date of interview (DD/MM/YYYY) | | | | | | | / / | | | |
| Name | | | (Given name) | | | | | | (Family name) | | | | | | (Other) | | |
| Date of birth | | | | | (Year) | | | | | (Month) | | (Day) | | | | Age |  |
| Place of residence | | | | | | |  | | | | | | | | | | |
| Did you immigrate into the present residence? | | | | | | | When? | | | | | | | | | | |
|  |  |  |  |  |  |  | Where did you live before your current residence? | | | | | | | | | | |
| Place of birth | | | | | |  | | | | | | | | | | | |
| Gender | | ❑ Female🡪  ❑ Male | | | | | | (If female)  Are you pregnant? | | | | | ❑ Yes ( months)  ❑ No | | | | |
|  |  |  |  |  |  |  |  | Are you lactating? | | | | | ❑ Yes  ❑ No | | | | |
| Highest education completed | | | | ❑ Postgraduate (S2, S3)  ❑ University (S1)  ❑ Diploma (S0 [D1–D4])  ❑ Senior High School (SMA)  ❑ Junior High School (SMP)  ❑ Elementary School (SD) or Less | | | | | | | | | | | | | |

**Question 1. We would like to know about your ethnic background**

Q1-1. What ethnic group do you belong to now?

|  |
| --- |

Q1-2. What ethnic group does your father belong to?

|  |
| --- |

Q1-3. What ethnic group does your mother belong to?

|  |
| --- |

Q1-4. What is your ethnic group in “three generations”?

|  |
| --- |

Note: Indonesian people usually recognize their ethnicity by genealogy (e.g., marriage between different ethnic groups) since their great-grandparents.

Q1-5. What language(s) do you speak (list all language(s) you can speak)?

|  |
| --- |

Q1-6. What language(s) does your father speak (list all language(s) your father can speak)?

|  |
| --- |

Q1-6. What language(s) does your mother speak (list all language(s) your mother can speak)?

|  |
| --- |

**Question 2. We would like to know about your religious background.**

Q2-1. What religion do you follow? Note: Your answer will be kept confidential.

❑ Islam ❑ Protestantism ❑ Catholicism ❑ Hinduism ❑ Buddhism ❑ Confucianism

❑ Others ( )

Q2-2. Do you follow local traditional religions to any extent?

❑ Yes (What is the name of the traditional religion you follow? )

❑ No

**Question 3. We would like to know about your occupational background**

Q3-1. What is your occupation?

|  |
| --- |

Q3-2. Do you/your household members engage in agriculture (are you a farmer)?

❑ Yes

If yes, do you/your household members sell the crops?

❑ Yes ❑ No

❑ No

Q3-3. Do you/your household members engage in fishing (including any methods)?

❑ Yes

If yes, do you/your household members sell the fish?

❑ Yes ❑ No

❑ No

Q3-4. Are you/your household members employed by company/government/others?

❑ Yes ❑ No

Q3-5. Do you/your household members run a business (e.g., store manager)?

❑ Yes ❑ No

q3-6. how many hours do you work on average per day?

❑ < 1 hour ❑ 1–2 hours ❑ 2–4 hours ❑ 4–6 hours ❑ 6–8 hours ❑ 8–10 hours ❑ < 10 hours

**Question 4. We would like to know your economic status**

Q4-1. How much did you earn in the last year?

|  |
| --- |

Q4-2. How much does your household earn on average every month?

❑ < Rp. 1.000.000 ❑ Rp. 1.000.000–2.000.000 ❑ Rp. 2.000.000–3.000.000

❑ Rp. 3.000.000–5.000.000 ❑ Rp. 5.000.000–7.500.000 ❑ Rp. 7.500.000–10.000.000

❑ > Rp. 10.000.000

❑ Do not know

**Question 5. We would like to know about your family environment.**

Q5-1. With whom do you live now?

❑ Alone

If you do not live alone, choose all

❑ Spouse

❑ Father

❑ Mother

❑ Spouse’s father

❑ Spouse’s mother

❑ Son/son-in-law (how many? )

❑ Daughter/daughter-in-law (how many? )

❑ Brother (how many? )

❑ Sister (how many? )

❑ Spouse’s brother (how many? )

❑ Spouse’s sister (how many? )

❑ Grandparents/spouse’s grandparents (how many? )

❑ Grandchildren/grandchildren-in-law (how many? )

❑ Other relatives (how many? )

❑ Fiancé /boyfriend/girlfriend

❑ Friends (how many? )

❑ Colleagues (how many? )

❑ Housekeeper/House servant (how many? )

❑ Others (how many? )

**Medical record (WRITTEN BY MEDICAL DOCTOR)**

Symptoms related to depression:

Diagnosis:

Start of symptoms:

Drug prescription:
