# Supplementary material for: Socio-economic-demographic determinants of depression in Indonesia: A hospital-based study
Source: PLoS One. 2020 Dec 15;15(12):e0244108. doi: 10.1371/journal.pone.0244108 (PMC7737985; doi:10.1371/journal.pone.0244108)
Supplement: Attachment — Submitted filename: Response to Reviewers.docx [file pone.0244108.s006.docx]

**Rebuttal Letter: Response to Academic Editor and Reviewer PLOS ONE**

Dear,

Antonio Palazón-Bru, PhD, Academic Editor, PLOS ONE

Prof. Byron Good, Dept of Global Health and Social Medicine, Harvard Medical School, Reviewer, PLOS ONE

We appreciate the opportunity to revise our manuscript entitled “Socio-economic-demographic determinants of depression in Indonesia: A hospital-based study”. Based on the helpful comments from the editor and reviewer, we have revised our manuscript extensively, which we feel has improved the quality of our work. We have carefully considered each comment and suggestion made by the editor and reviewer. Below we present our responses to the editor, followed by our responses to the reviewer. The comments of the editor and reviewer are shown in italics, with our responses shown in bold text.

Response to Editor’s comments

1. *Please ensure that your manuscript meets PLOS ONE's style requirements, including those for file naming. The PLOS ONE style templates can be found at*

[*https://journals.plos.org/plosone/s/file?id=wjVg/PLOSOne_formatting_sample_main_body.pdf*](https://journals.plos.org/plosone/s/file?id=wjVg/PLOSOne_formatting_sample_main_body.pdf)  *and*

[*https://journals.plos.org/plosone/s/file?id=ba62/PLOSOne_formatting_sample_title_authors_affiliations.pdf*](https://journals.plos.org/plosone/s/file?id=ba62/PLOSOne_formatting_sample_title_authors_affiliations.pdf)

**We carefully checked our manuscript and made corresponding changes to meet the journal’s style requirements.**

2. *Please include additional information regarding the survey or questionnaire used in the study and ensure that you have provided sufficient details that others could replicate the analyses. For instance, if you developed a questionnaire as part of this study and it is not under a copyright more restrictive than CC-BY, please include a copy, in both the original language and English, as Supporting Information*

**We have included our interview form in both languages as supporting information, labelled as S5 in the Appendix.**

Response to Reviewer’s comments

*This study compares a sample of persons being treated in hospital-based psychiatric outpatient clinics in Makassar, Indonesia, with a matched randomly selected population of persons living within a 30 minute walk of the university hospital. The study is important for two reasons. First, there is so little research on depression in Indonesia (the 4th most populous nation in the world), compared with studies of depression in most societies in the world, and compared with studies of psychotic illness in Indonesia. It is thus very welcome to find a study examining basic characteristics of persons being treated for major depressive disorder in a psychiatric outpatient setting Makassar. Second, the study is important because the most crucial finding of the study -- that persons being treated for depression in this sample has higher income than in the control population -- is unexpected and counterintuitive. It thus raises important questions for further study, suggesting in particular the need for further population-based studies in Indonesia, as well as studies of care-seeking patterns and access to care for persons with depressive illnesses* *(paragraph 1)*

*I appreciate the hypotheses the authors considered in explaining why this treated population has higher income than the comparison population. These are important hypotheses that should indeed generate future studies. And the suggestion that the public health services need to focus more attention on identifying and treating depression among lower income groups is an important one -- and suggests further studies of how best to do this (paragraph 3)*

**We thank the reviewer for their positive comments regarding our research.**

*There is a great deal of focus on methodology, in particular methods for dealing with missing values (particularly for individual income), in this study. Overall, I leave it for others to discuss the statistical procedures. However, I do wonder why the study gathered information about individual income rather than household income. Because wealth and social status accrue to households, not individuals, particularly in Indonesia, there may be conflation of income and gender (although number of 'housewives' is quite low).*

**Household income is appropriate for measuring an individual’s household wealth and social status in society, but does not necessarily reflect their personal social status. In addition, household income is not always known by all household members (e.g., a patient with depression patient may not be aware of the income of other household members).**

**Personal income is known to influence the specific opinions and decisions of individuals (Kuhn, 2019)^[1]^. According to Ross and Huber (1985)^[2]^:**

**“Personal earnings, by adding to family income, decrease economic hardship and depression. But personal earnings, independently of their purely financial aspects, may also affect depression. Family (or households) income, from whatever source – a spouse's earnings, social security, or public assistance – is important to psychological well-being because it allows one to pay the bills, and feed, clothe, and care for the health of one’s family. But the money one earns oneself may affect well-being in other ways (p. 315)”.**

**In addition, the introduction section of our manuscript describes a similar issue:**

**“The mechanism (of income cause depression) may be explained absolutely by one’s ability to secure basic needs (e.g., in purchasing goods and services), including health, or relatively by implying social position and emotional satisfaction in terms of SES producing happiness (p. 3; line 59-62)”.**

**Thus, we felt that it was better to gather information about personal income than household income to understand better how people think or decide (absolute vs. relative), and how their income affects their mental health. However, we thank the reviewer for raising this point, and we apologize that we did not describe this issue clearly in the previous version of the manuscript. We have addressed this issue in more depth in the revised discussion section (p. 16-17; line 271-278).**

*One other methodological issue -- in selecting the comparison population. It seems that data were not collected about where the patient population resides in Makassar, and whether the neighborhood "within a 30 minute walk" is indeed a good comparison. We know that major cities in Indonesia are stratified by neighborhoods. It would be useful for the authors to add a note to indicate whether the neighborhoods around the university hospital have particular characteristics and whether most of the patients come from this same area.*

**In the discussion section, we described the difficulty of matching case and control participants as a limitation. However, in the previous version of the manuscript, we did not explain why we chose participants from communities “within a 30-minute walk” for comparison with the case participants, which represents a potential limitation of the current study. We initially attempted to recruit control participants in a way that closely matched the neighborhood characteristics of the case group; however, most individuals refused to participate because of difficulties related to distance. As an alternative method, we searched communities that were located close to the hospital, which we assumed would have similar characteristics to the case group. Nevertheless, we agree that this issue should be clearly discussed as a limitation in the manuscript. Thus, we have added an explanation to address this issue in the revised discussion section (p. 14-15; line 232-236).**

*This then raises one critical issue that should be added to the discussion session. In what ways do persons from lower SES groups experience and express major depression, and what are the common pathways for care-seeking among these groups? There is remarkably little research on this topic in Indonesia, compared with many parts of the world. However, as an anthropologist who has worked on issues of mental illness in Indonesia for years, I would say that symptoms of depression are often not recognized as a treatable medical condition among many persons in lower socioeconomic and lower educated groups. To the extent they are viewed as illness, they are often treated in the popular and religious healing sectors rather than conceived as matters for treatment through medical settings. The primary health care system has recently been mandated to provide care for persons with severe mental illness in Indonesia, but there is no mandate to recognize and treat depression. Rates of recorded depression in medical records in the public primary health clinics are extremely low. My point is simply that in the discussion session, where the authors consider critical hypotheses for explaining an important unexpected finding, this set of issues should be recognized and put on the agenda for future research.*

*Overall, I find this an important paper that with minor revisions should be published.*

**We thank the reviewer for raising this critical issue about how people with lower incomes experience and express depression, and their common care-seeking pathways. We agree with the reviewer’s suggestion regarding the possible explanations of this issue, and have elaborated on this point in the discussion section (p. 15-16; line 251-260). Furthermore, we realize that it is important to consider a critical hypothesis for explaining this important and unexpected finding. Therefore, we modified our discussion of this point at the end of the discussion section. With Professor Good’s permission, we would like to cite the reviewer’s comment itself as personal communication with Professor Byron Good, because we feel that this insight from an anthropological perspective is valuable.**

Once again, we thank the editor and reviewer for their time and effort in reviewing our manuscript, which we feel has been much improved as a result.

**Yours sincerely,**

**Andi Agus Mumang**

1. Kuhn, U. (2019). Measurement of income in surveys. FORS Guide No. 02, Version 1.0. Lausanne: Swiss Centre of Expertise in the Social Sciences FORS. doi:10.24449/FG-2019- 00002
2. Ross CE, Huber J. Hardship and depression. J Health Soc Behav. 1985;26(4):312–27. Availabe at: https://www.jstor.org/stable/2136655
